# The Influence of Digital Democracy, Political Literacy, and Post-Truth Politics on Gen Z Political Participation in the 2024 Elections in Indonesia

**DOI:** 10.12688/f1000research.185707.1

**Published:** 2026-07-13

**Authors:** Agust Jovan Latuconsina, Fendy Suhariadi, Suparto Wijoyo, Ian Firstian Aldhi

**Affiliations:** 1 Universitas Airlangga, Surabaya, East Java, Indonesia

**Keywords:** Digital Democracy; Political Literacy; Post-truth Politics; Political Participation; Gen Z

## Abstract

**Background:**

Digital democracy has transformed political communication and participation, especially among Generation Z voters who actively use social media and digital platforms. In the 2024 Indonesian election, digital spaces became important arenas for accessing political information, expressing opinions, and engaging in public debate. However, the rise of post-truth politics also created challenges through misinformation, disinformation, and emotionally driven political narratives.

**Methods:**

This study used a quantitative survey design to examine the influence of digital democracy, political literacy, and post-truth politics on Generation Z’s political participation in the 2024 Indonesian election. The respondents were Generation Z voters aged 18–28 years who were registered in the 2024 Permanent Voter List across 38 provinces in Indonesia. Data were collected using a structured questionnaire with a four-point Likert scale. The data were analyzed using Structural Equation Modeling (SEM) with AMOS to test direct, mediating, and moderating relationships among the variables.

**Results:**

The findings show that digital democracy significantly strengthens Generation Z’s political literacy. Political literacy also has a significant positive influence on political participation. In addition, digital democracy directly influences political participation and indirectly influences it through political literacy. This indicates that political literacy functions as a mediator between digital democracy and political participation. The study also finds that post-truth politics negatively moderates the relationship between political literacy and political participation, meaning that the positive effect of political literacy becomes weaker when post-truth politics is high.

**Conclusions:**

Digital democracy and political literacy are important factors in strengthening Generation Z’s political participation in the 2024 Indonesian election. Political literacy helps Gen Z voters evaluate political information critically and participate more meaningfully, while post-truth politics can weaken this process through biased and manipulative information.

## Introduction

Several studies have simultaneously examined the influence of digital democracy and political literacy on Gen Z’s political participation, with post-truth politics as a moderator in the context of the 2024 elections in Indonesia. As the largest group of voters, Gen Z is a voting bloc with its own segmentation in voting projections.

Indonesia has experienced several periods, with reforms being a political period that continues to this day. The existence of political parties is an inevitable political reality as a pillar of democracy and exercise of people’s sovereignty. Political parties have a task framework, namely, a central and important position (status) and role in every democratic system because they play the role of a link between the state government and its citizens ( Vílchez & Achmad, 2023).

The five-yearly democratic celebration, which should be an opportunity to demonstrate the progress of Indonesian civilization, has become a ‘nightmare’ for the people, with massive amounts of hoaxes, propaganda, misleading opinions and hate speech making it seem as if the nation no longer regards Pancasila as the philosophy of national and state life.

The dynamics of the 2024 elections have also utilized digital instruments in Indonesia’s political processes. This has resulted in digital democracy, while also influencing the democratic process and becoming embedded in the closest layers of people’s social lives. This has added to the complexity of national life during this period, so it seems as if this extraordinary polarization has been designed to endanger national security due to the influence of social media ( Badrun, 2020).

The existence of digital media leaves a paradoxical situation between having a positive impact and causing deep wounds to the current state of democracy. After the collapse of the Soeharto regime, the media and information were under full government control. Indonesia has entered a new phase through technological penetration along with the growth and development of new information media as a means of public reporting. Although government policy through the Electronic Information and Transactions Law has stifled citizens’ freedom of opinion and expression, it has caused the maturation of democracy to stagnate over the past five years.

The tide of globalization cannot be stemmed, and digitalization has demonstrated its existence in humans as political beings and variables of democracy. The fundamental values of democracy, freedom/liberty, have made individuals free to do anything in their role as citizens who have the right to criticize or even counter-argue government decision-making, for example, in the process of drafting legislation, or even citizens who are free to choose anyone to represent them in parliament.

Over the past decade, internet access in Indonesia has experienced tremendous growth. This shows that more Indonesians are connected to the internet. By 2024, more than 221 million Indonesians will use the internet, covering approximately 79.5% of the country’s population. Since 2018, Indonesia’s internet penetration has reached 64.8%. Subsequently, it reached 73.7% in 2020, 77.01% in 2022, and 78.19% in 2023. Based on gender, Indonesia’s Internet penetration rate comes mainly from men (50.7%) and women (49.1%). In terms of age, most people surfing the Internet were Gen Z (born between 1997 and 2012), accounting for 34.40%. There are several forms of hoaxes that emerged ahead of the 2024 elections, such as posts on Facebook, claiming that the 2024 General Election has been postponed due to ethical violations reported by the Election Organizers Honorary Council (DKPP), and the presidential candidate pair–vice presidential candidate pair number 2–Prabowo Subianto–Gibran Rakabuming Raka–had been disqualified. In addition, posts circulating on Facebook via chain messages stated that the General Election Commission (KPU) would no longer be sending physical invitations to vote in the 2024 General Election. During the 2024 elections, hoaxes continued to spread in Indonesia.

According to Dunan (2019), social media has changed the way in which government public relations communicate. The use of new media such as Facebook, Twitter, and Instagram can increase interactions between netizens and the government. This digital space can increase public participation in expressing their aspirations while also indicating the government’s efforts to communicate openly, interactively, and participatively.

At the beginning of 2024, the number of Internet users in Indonesia reached 79.5% or 221.6 million people. The use of mobile connections has increased by 3.6% since the COVID-19 pandemic began. Currently, fiber optic penetration has reached 60.84% in all sub-districts. The fiber-optic network covers approximately 4,398 sub-districts out of a total of 7,281 sub-districts in Indonesia. The total length of the fiber-optic network built to date is 460,000 km. In several provinces of Indonesia, mobile broadband download speeds exceed 25 Mbps. Currently, in the 5G era, 5G network penetration is already present in 48 cities. Broadband network penetration increases data usage and internet traffic. This, in turn, leads to a continuous increase in the need for data centers, servers, and cloud services ( Kementerian Komunikasi dan Digital, 2024).

Public discourse in Indonesia’s digital space is frequently shaped by policy issues initiated by the government, such as COVID-19 travel requirements and quarantine rules for international arrivals ( Sekretariat Kabinet Republik Indonesia, 2021, 2022), as well as debates surrounding the Omnibus Law on Job Creation ( Marpaung, 2022). These issues have generated varied and often opposing reactions from citizens. On the one hand, dialogue among citizens in digital spaces can support democratic deliberation. On the other hand, the digital public sphere is also vulnerable to hoaxes, fake news, and post-truth dynamics, in which objective facts become less influential than appeals to emotion and personal belief in shaping public opinion ( Oxford Languages, 2016; Utomo, 2022; Arifin & Fuad, 2021). The behavior of Indonesian society, which actively interacts through social media to meet its information needs, often comes face-to-face with fake news that is sometimes difficult to digest and distinguish between what is true and what is manipulative ( Kwanda & Lin, 2020).

According to Loveless (2020), the phrase ‘Fake news as an emotional weapon’ represents the meaning of the post-truth phenomenon itself and how these variables live and stick in the digital space in Indonesia. Therefore, it is natural for some observers, academics, and netizens in this republic to view hoaxes, fake news, and their ‘accomplices’ as being fabricated by certain parties for a specific purpose. Post-truth is the relativization of truth with data objectivity, where the dramatization of a message is far more important than the content of the message itself ( Reali, 2026). The consumption of news and information from digital media is always adorned with dramatic and bombastic headlines as if they fully reflect the substance of the message being conveyed. Fake news, propaganda, hoaxes, misinformation, and hate speech would not exist in the digital space if every citizen truly understood the essence of the Pancasila democracy, which was championed by their predecessors.

According to Dragolea et al. (2023), Gen-Z behavior can be grouped into four major components based on one strong foundation: Gen-Z is a generation that seeks truth. First, Gen-Z is ‘The Undefined ID,’ where this generation values the expression of everyone without labeling them. Second, The Communaholic generation is very inclusive and interested in getting involved in various communities by utilizing technological sophistication to expand the benefits they want to provide. The third is activism; they can be mobilized for various social and environmental issues and have a high concern for others and the world around them, while the fourth is their analytical and pragmatic attitude, and they often question rules and authority.

This study discusses three main variables related to Gen Z’s political participation in Indonesia. These three variables include digital democracy, political literacy, and post-truth politics. These three variables were measured through a survey conducted in Indonesia. Furthermore, this study is interesting because, to the best of our knowledge, no study has discussed these three variables simultaneously.

The novelty of this study relates to the three main variables that influence Gen Z’s political participation: digital democracy, political literacy, and post-truth politics. There is still very little research that uses post-truth politics as a moderating variable to analyze its relationship with Gen Z’s political participation. Many studies have been partially conducted on the four variables, and some have linked one variable to another. For example, research has analyzed the influence of digital democracy on Gen Z’s political participation. Research on political literacy and its influence on Gen Z’s political participation. This study was conducted to analyze the influence of digital democracy on Gen Z’s political participation in Indonesia amid rapid changes in information technology and the use of digital instruments. Social media has played an important role as the main instrument for political information channels in the community. Thus, Gen Z in Indonesia has a good political preference for understanding political platforms, political opinions, and political developments quickly, impartially, and transparently.

## Theoretical framework and hypothesis

### 2.1 Digital democracy

Some observers of cyberspace have assumed that the internet is inherently democratic. However, Diaz (2008) argues that this assumption should be critically examined, particularly because search engines and digital platforms may reproduce sociopolitical biases. At the same time, internet-based communication can provide new spaces for political information, public expression, and civic participation through online public spheres, blogs, social networking sites, citizen journalism, and other forms of online political communication ( Dahlgren, 2005; Shah et al., 2005; Best & Krueger, 2005). E-democracy refers to the use of information and communication technology (ICT) in political debates and decision-making processes, either complementing or contrasting traditional forms of communication, such as face-to-face interaction or one-way mass media ( Päivärinta & Sæbø, 2006). Similarly, digital democracy can be understood as the pursuit and practice of democracy through digital media in both online and offline political communication ( van Dijk, 2012). In the Indonesian context, Manggalanny describes digital democracy as the collective use of digital media and networks for political purposes, whether online, offline, or in combination ( Kompas.com, 2021).

E-democracy and digital democracy are closely related to public access to information, online political communication, e-participation, e-deliberation, transparency, and public accountability. These practices may include digital platforms for consultation, petitions, campaigns, polling, deliberative forums, and access to open government data ( Council of Europe, 2009; Lindner & Aichholzer, 2019). The internet can extend and pluralize the public sphere by creating new spaces for political information, discussion, and civic engagement, although these spaces do not automatically guarantee deliberative quality or equal participation ( Dahlgren, 2005; van Dijk, 2012).

Digital democracy also enables political messages to circulate quickly and widely through social media, websites, and digital applications, facilitating communication among governments, political parties, activists, and citizens. It may encourage civic participation in discussions, campaigns, social movements, and public monitoring. However, digital democracy also faces challenges, including misinformation, disinformation, echo chambers, algorithmic bias, political surveillance, and unequal digital access ( Lindner & Aichholzer, 2019; OECD, 2024). Therefore, digital democracy should be understood not only as the expansion of online participation, but also as a democratic process that requires information integrity, digital literacy, transparency, accountability, and protection of citizens’ rights.

### 2.2 Political literacy

Political literacy refers to citizens’ knowledge and understanding of political processes and issues, enabling them to perform their roles effectively and participate in democratic life ( Denver & Hands, 1990). It also involves practical understanding of political concepts drawn from everyday life, including the ability to understand political issues, the positions of political actors, and how these issues may affect oneself and others ( Crick, 2000). In this sense, political literacy is not limited to factual knowledge, but also includes citizens’ capacity to evaluate political information, understand democratic procedures, and make informed political decisions.

Previous studies show that education and political knowledge are closely related to political engagement. Cassel and Lo (1997) explain that political literacy can be examined through cognitive mobilization, socialization, and structural factors, while Campbell et al. (1960) and Converse (1964) emphasize that political awareness and ideological understanding are shaped by citizens’ level of political information and political socialization. Similarly, Delli Carpini and Keeter (1996) argue that politically informed citizens are more likely to participate, recognize their own interests, and advocate those interests through political action.

The absence of political literacy, particularly among first-time voters, may create several socio-political problems. Voters with low political literacy are more vulnerable to political propaganda, misinformation, vote buying, political apathy, and manipulative campaign strategies ( Karim et al., 2015; Sutisna, 2017; Tam, 2016). In the digital context, social media can support political literacy by providing access to political information and public discussion, but it can also expose novice voters to misleading political messages and online misinformation ( Ohme, 2019; Kurniasih et al., 2024). Therefore, strengthening political literacy is important for encouraging rational, informed, and independent political participation, especially among young and first-time voters ( Pontes et al., 2019; Dudley & Gitelson, 2002).

### 2.3 Post-truth politics

Post-truth politics can be understood as a form of political discourse that is epistemically dysfunctional because it no longer places objective facts as the main basis for shaping public opinion and political decision-making. Oxford Languages defines *post-truth* as circumstances in which objective facts are less influential in shaping public opinion than appeals to emotion and personal belief ( Oxford Languages, 2016). Rietdijk (2024) argues that post-truth politics does not only damage knowledge and democracy, but may also weaken citizens’ epistemic autonomy through counter-narratives, the discrediting of criticism, and the denial of relatively clear facts. In a similar sense, Keyes (2004) describes the post-truth era as a condition in which the boundaries between truth and lies, honesty and deception, as well as fact and fiction, become increasingly blurred.

In a democratic system, freedom of speech is an essential requirement. However, such freedom still requires a commitment to facts. Without shared facts, deliberation, negotiation, and democratic decision-making can easily turn into a contest of emotions and group-based justification. Arendt (1967) distinguishes between rational truth and factual truth. Factual truth is important in politics because it concerns events and realities that become the basis of public debate. Therefore, truth cannot be separated from democracy, especially in a society marked by polarization, internet penetration, social media use, and the rapid spread of unverified information.

The phenomenon of post-truth becomes stronger in the digital sphere because of information manipulation. Giusti and Piras (2021) explain that disinformation, fake news, and post-truth politics have become serious challenges to democracy because they transform the information landscape and weaken democratic foundations. Al-Rodhan (2017) argues that post-truth politics relies more on emotion and intuition than on facts and evidence. Fake news and conspiracy theories can spread quickly, create alternative realities, and be used for propaganda purposes. In the construction of discourse, Hamad (2017) identifies three important elements: framing, signing, and priming. Framing refers to the selection of issues, actors, storylines, arguments, risks, and responsibilities. Signing refers to the selection of signs, such as words, symbols, images, slogans, colors, and order of presentation. Priming refers to the effort to ensure that a discourse reaches the audience by increasing the opportunity to access, read, remember, and share the message.

The main characteristics of post-truth politics include provoking public emotions, ignoring data and facts, spreading political news whose truth has not been verified, combining populist movements with conspiracy theories, mobilizing fictional narratives about certain figures or events, and polishing dishonesty in order to strengthen the social, political, or economic position of particular actors or groups. Arifin and Fuad (2020), referring to Fahruddin Faiz, explain that the roots of post-truth politics include the human desire for personal identity reinforcement, media orientation toward ratings, clicks, followers, and profit, the commodification of information, technological progress that is not accompanied by social intelligence, short-term pragmatism, a society that is easily attracted to sensational issues, and populism that uses rhetoric such as “for the people” or “defending the people” for specific political interests.

Post-truth politics is also closely related to hoaxes and fake news. Allcott and Gentzkow (2017) define fake news as news articles that are intentionally and verifiably false and that may mislead readers. They distinguish fake news from unintentional reporting errors, rumors, conspiracy theories that are difficult to verify, satire, false statements by politicians, and biased reports that are not entirely false. In UNESCO’s framework, information disorder is divided into misinformation, disinformation, and malinformation. Misinformation refers to false information shared without the intention to cause harm. Disinformation refers to false information deliberately created and shared to deceive. Malinformation refers to genuine information that is used in a particular context to harm a person, group, organization, or country. The motives behind the spread of hoaxes may include political, economic, ideological, sentimental, or personal interests, and sometimes merely entertainment or the desire to gain attention.

### 2.4 Political participation

Political participation refers to citizens’ involvement in political life through activities such as voting, joining political parties, participating in pressure groups, engaging in campaigns, or taking part in actions aimed at influencing government and public policy. Leach, Coxall, and Robins (2006) describe political participation as citizen involvement in politics through activities such as voting, pressure group activity, and party activity directed toward influencing government and public policy. Ruedin (2007) further explains that political participation is an individual activity, although it is carried out within a particular institutional and social context and often involves interaction with others. Similarly, McClosky (1968) defines political participation as voluntary activities through which members of society take part in selecting rulers and, directly or indirectly, in forming public policy. Huntington and Nelson (1976) define political participation as activity by private citizens designed to influence government decision-making.

In a democratic system, political participation is important because democracy allows citizens in a pluralistic society to express their interests, preferences, and aspirations. However, participation does not always produce healthy democratic deliberation. When citizens participate mainly through partisan affiliation, emotionally charged narratives, and selective information environments, democratic participation can become trapped in an echo chamber. In this condition, individuals are repeatedly exposed to information that confirms their existing beliefs, while opposing perspectives are rejected or ignored. When echo chambers are combined with post-truth politics, political participation may strengthen partisanship, deepen polarization, and reduce the quality of public dialogue.

Participatory citizenship is broader than formal political participation. It includes actions through which individuals contribute to public life, community well-being, and shared social purposes. The Civic Engagement VALUE Rubric developed by the Association of American Colleges and Universities and edited by Rhodes (2010) explains that civic engagement involves actions in which individuals participate in activities of personal and public concern that are both personally meaningful and socially beneficial. Participatory citizenship can be understood through several dimensions. First, civic identity refers to the ability to see oneself as an active participant in society, with responsibility to work with others toward public purposes. Second, civic communication refers to the ability to listen, deliberate, negotiate, build consensus, and use conflict productively. Third, civic action and reflection refer to the ability to show initiative in civic leadership and to reflect critically on the goals and outcomes of one’s civic actions.

The level of political participation is influenced by several factors. The civic voluntarism model developed by Brady, Verba, and Schlozman (1995) explains that participation is shaped by resources, political engagement, and recruitment networks. Resources include time, money, and civic skills. Political engagement includes political interest, information, efficacy, and motivation. Recruitment networks refer to the social and organizational connections through which individuals are invited or encouraged to participate. In this sense, socio-economic status, education, age, political interest, partisanship, and membership in organizations or pressure groups can all affect the extent and form of a person’s political participation.

Political participation can also be discussed through different forms and purposes. Informational participation refers to activities aimed at spreading political information, educating citizens, and increasing public awareness of political issues. Norris (2002) shows that political activism has expanded beyond traditional electoral participation and now includes newer forms of civic and political engagement, including those connected to media, protest, and the internet. Examples of informational participation include sharing verified political information on social media, organizing seminars about citizens’ rights, and creating educational content about democratic processes.

Strategic participation refers to systematic and collective forms of participation aimed at producing political or policy change. Tilly (2004) explains that social movements are vehicles of popular politics that use campaigns, repertoires of action, and public displays of worthiness, unity, numbers, and commitment to make collective claims. Examples of strategic participation include mass movements demanding political reform, alliances among civil society groups to push constitutional amendments, and evidence-based advocacy campaigns aimed at changing public policy.

Instrumental participation refers to participation used as a means to achieve particular objectives. White (1996) explains that participation may serve different interests and should always be understood politically because it can either challenge unequal power relations or reproduce them. White’s model distinguishes nominal, instrumental, representative, and transformative participation. Instrumental participation is often used to achieve practical or short-term goals, while transformative participation aims to change structures of power and create deeper forms of empowerment. Therefore, political participation should not only be measured by how many citizens participate, but also by the quality, purpose, inclusiveness, and democratic impact of their participation.

### 2.5 The generation Z

Generation Z refers to a generational cohort that grew up in an environment shaped by the internet, social media, mobile technology, and digital communication. Singh and Dangmei (2016) describe Generation Z as a generation raised with the social web, in which digital technology becomes central to identity, communication, and everyday life. Francis and Hoefel (2018) define Generation Z broadly as those born between 1995 and 2010 and describe them as “true digital natives” because they have been exposed to the internet, social networks, and mobile systems from an early age. However, the birth-year boundary of Generation Z is not universally fixed. For example, Dimock (2019) explains that Pew Research Center uses 1996 as the final birth year for Millennials, meaning that those born from 1997 onward are categorized as part of the next generation.

The concept of generation can be understood sociologically through Mannheim’s theory of generations. Mannheim (1952/1928) argues that a generation is not only a group of people born within the same period, but also a social location shaped by shared historical experiences. In other words, people in the same generation may develop similar attitudes because they encounter important social, political, cultural, and technological events during the same stage of life. Ryder (1965) also explains that cohorts are shaped by shared historical experiences, peer-group socialization, and changes in education and society. Putra (2016) further explains that generational differences emerge because individuals who are born in a similar period tend to experience similar social and historical conditions.

Generation Z is often described as the first generation that truly grew up with the internet as part of daily life. Grail Research (2011) states that Generation Z is the first real internet generation. Unlike Generation Y or Millennials, who experienced the transition into digital technology, Generation Z was born when digital technology was already widely available. This condition shapes several characteristics of Generation Z, including technological fluency, flexibility, global connectedness, openness to cultural differences, and strong dependence on virtual networks. At the same time, this generation is also often associated with instant culture, high exposure to online information, and lower sensitivity toward privacy because many aspects of their lives are shared through social media.

The digital environment also makes Generation Z highly expressive and socially connected. Sladek and Grabinger (2014) argue that Generation Z tends to respond actively to experiences, whether positive or negative, by expressing them through digital platforms. This means that Generation Z does not only consume information, but also produces, shares, and comments on information in public digital spaces. In this sense, Generation Z can become influential in society because their opinions, experiences, and reactions can circulate quickly through social media networks.

Tulgan (2013) identifies several formative factors that shape Generation Z. These include the central role of social media, the importance of human connection, the skills gap, a global mindset, and local realities. These factors show that Generation Z is both globally connected and locally situated. They can access global information, trends, and communities through digital platforms, but their attitudes and choices are still shaped by local social, economic, cultural, and political conditions.

In the Indonesian context, Wibawanto (2018) identifies several characteristics of Generation Z. First, they are technologically fluent because they are familiar with digital devices, applications, and online platforms. Second, they are highly connected through social media and often use it as a space for communication, self-expression, and social interaction. Third, they tend to be expressive in presenting opinions, identities, and experiences. Fourth, they are fast switchers, meaning that they can move quickly from one idea, task, or activity to another. Linnes and Metcalf (2017) also note that younger generations face a digital environment filled with technological options and distractions, which affects how they interact with information and learning media.

Therefore, Generation Z can be understood as a digital-native generation whose identity, social interaction, learning style, and public expression are strongly influenced by internet culture and social media. Their familiarity with technology gives them advantages in accessing information, building networks, and participating in public discourse. However, it also creates challenges related to privacy, attention, information quality, and the tendency to respond quickly before verifying information.

### 2.5 Hypothesis development

*2.5.1. The Relationship Between Digital Democracy (DD) and Political Literacy (PL)*

Digital democracy (DD) refers to the use of digital information and communication technologies to expand citizens’ access to political information, public deliberation, and political participation. It includes the use of social media, civic technology platforms, online consultation tools, digital public forums, and other internet-based mechanisms that enable citizens to obtain information, express opinions, monitor government, and participate in democratic processes. Congge et al. (2023) explain that digital democracy provides a new space for community involvement in democratic life and shows that public knowledge is an important condition for quality digital democracy. Similarly, Saud et al. (2023) argue that social media functions as a platform for political communication, political expression, and digital democracy, while Weinhardt et al. (2024) emphasize that literacy, inclusion, and transparency are essential pillars for resilient digital democracies.

Political literacy (PL) can be understood as the ability of citizens to understand political institutions, political processes, public issues, democratic values, and their own role in civic and political life. It is not limited to political knowledge, but also includes critical thinking, democratic attitudes, political self-efficacy, and the willingness to participate in public decision-making. Schmitt et al. (2024) explain that civic education in digital environments aims to foster political knowledge, positive attitudes toward democracy, political self-efficacy, and willingness to participate in the political process. Prasetiyo et al. (2025) also highlight the importance of civic literacy in democratic learning, especially in the digital era, where citizens need the ability to understand, evaluate, and participate in democratic life.

Theoretically, digital democracy can strengthen political literacy because digital platforms provide citizens with continuous access to political news, policy debates, candidate messages, public criticism, and civic education content. Through digital spaces, citizens are not only passive recipients of political information, but also active participants who can discuss, contest, reinterpret, and share political messages within interactive networks. Repeated exposure to political information and online deliberation can improve citizens’ understanding of political issues, actors, institutions, and democratic procedures. Boulianne (2020), through a meta-analysis of digital media effects, shows that digital media use is generally associated with civic and political engagement. Dahlgren (2013) also argues that the political web creates new possibilities for political participation, civic subjectivity, and alternative democratic engagement.

Empirical studies further support the relationship between digital democracy and political literacy. Satria et al. (2023) found that social media plays an important role in increasing the political literacy of first-time voters by disseminating information about political processes, development policies, public opinion, and democratic control. Efebeh et al. (2024) also found that social media has a positive and statistically significant effect on the political literacy of first-time and eligible voters in rural communities in Nigeria. These findings indicate that social media, as one of the main arenas of digital democracy, can improve citizens’ political awareness, political knowledge, and engagement with electoral issues.

However, the relationship between digital democracy and political literacy is not automatically positive. Digital democracy can also expose citizens to misinformation, disinformation, hate speech, algorithmic bias, and manipulative political content. Internews (2023) notes that electoral disinformation can harm democratic institutions and public confidence, and therefore recommends improving digital knowledge and coordinated strategies among governments, technology companies, and civil society. Weinhardt et al. (2024) similarly argue that citizens need the skills to navigate, criticize, and contribute to the digital sphere. Therefore, digital democracy is more likely to strengthen political literacy when citizens have sufficient digital literacy, critical awareness, access to credible information, and opportunities for meaningful deliberation.

Based on this theoretical and empirical explanation, digital democracy is expected to positively influence political literacy. The more citizens engage with credible, participatory, and deliberative digital democratic spaces, the more likely they are to develop political knowledge, democratic attitudes, political efficacy, and willingness to participate in civic and political life. Accordingly, we propose the following hypothesis:

Hypothesis 1 (H1). *Digital Democracy positively influences Political Literacy.*

*2.5.2. The Relationship Between Political Literacy (PL) and Political Participation (PP)*

Political literacy can be understood as a multidimensional competence that combines political knowledge, interpretive skills, critical awareness, and civic dispositions. It enables citizens to understand political institutions, electoral procedures, public policy issues, democratic values, and their own rights and responsibilities in political life. Eriksen (2020) emphasizes the importance of political literacy as a capacity to understand and assess political processes, while civic education literature commonly links political literacy with citizens’ ability to participate effectively in public life. In this sense, political literacy is not only concerned with knowing political facts, but also with the ability to interpret political information, evaluate political claims, and make informed political choices.

Political participation refers to citizens’ activities that are intended to influence political decision-making, government action, or public policy. These activities may include voting, joining campaigns, participating in political discussions, contacting public officials, joining interest groups, signing petitions, engaging in demonstrations, or using digital platforms to express political opinions. Political literacy is theoretically important for participation because it reduces cognitive and psychological barriers to engagement. Citizens who understand their rights, electoral procedures, party platforms, public issues, and available channels of influence are more likely to see political participation as meaningful and possible.

The relationship between political literacy and political participation can be explained through several mechanisms. First, political literacy increases political knowledge, allowing citizens to understand political issues and institutional processes more clearly. Second, political literacy strengthens internal political efficacy, namely the belief that one is capable of understanding and participating in politics. Third, political literacy helps citizens evaluate political information more critically, reducing their vulnerability to misinformation, clientelism, and emotional manipulation. Fourth, political literacy clarifies the benefits and consequences of political engagement, thereby increasing citizens’ motivation to participate.

Empirical studies support this relationship. Revalina et al. (2024), in a study of first-time voters in Kerinci Regency before the 2024 Indonesian election, found a significant positive relationship between political literacy and political participation. Their study measured political literacy and political participation among novice voters and showed that higher political literacy was associated with greater participation before the election. Similarly, Sutanto (2024), in a descriptive study of PPKn students at STKIP Pasundan, found that political literacy obtained through political science courses, lectures, presentations, discussions, literature studies, focus group discussions, field studies, and assignments helped students understand political information and encouraged participation in the 2024 simultaneous regional elections.

Evidence from voter education studies also strengthens this argument. Noprianto, Wantu, and Djaafar (2025) found that political education programs in Banggai Regency were associated with improved voter participation in the 2024 general election. The study shows that voter education programs contributed to an increase in voter turnout from 79.47% in the 2019 election to 80.83% in the 2024 election. It also notes that limited cognitive, affective, and evaluative abilities, limited access to information, and economic instability can become obstacles to political participation. This suggests that political literacy is important not only for increasing the quantity of participation, but also for improving the quality of participation.

At a broader level, research on civic education provides additional support for the link between political literacy and participation. Willeck and Mendelberg (2022) explain that the relationship between education and political participation is complex and that causal evidence is mixed. However, they also show that how civic education is taught matters, especially when it uses active-learning strategies. Weinberg (2022) similarly found that civic education in English secondary schools may increase young people’s expressive political participation and future voting intentions, particularly when students experience open classroom climates and participatory learning. These findings indicate that political literacy is more likely to encourage participation when it is developed through active, discussion-based, and reflective learning processes.

Therefore, political literacy can be considered a proximal determinant of political participation. Citizens who are politically literate are more likely to understand political issues, feel capable of participating, evaluate political information critically, and use available democratic channels to express their interests. Based on this theoretical and empirical explanation, this study proposes the following hypothesis:

Hypothesis 2 (H2). *Political Literacy positively influences Political Participation.*

*2.5.3. The Relationship Between Digital Democracy (DD) and Political Participation (PP)*

Digital democracy refers to the use of digital technologies to expand citizens’ opportunities to access political information, deliberate on public issues, interact with political actors, and participate in decision-making processes. It includes online consultations, social media campaigns, civic technology platforms, e-government services, digital petitions, participatory budgeting platforms, and other forms of e-participation. Asimakopoulos, Antonopoulou, Giotopoulos, and Halkiopoulos (2025) explain that information and communication technologies influence democratic processes and citizen participation through tools such as social media, e-voting systems, e-government initiatives, and e-participation platforms. These tools can improve the quality, efficiency, and transparency of democratic engagement, although they also create challenges related to privacy, misinformation, digital divides, and security.

In digital democratic environments, citizens can engage in relatively low-cost political activities, such as sharing political content, signing online petitions, joining digital public discussions, participating in online consultations, following campaign information, and interacting directly with representatives. These activities reduce participation barriers related to time, distance, access to information, and organizational resources. Therefore, digital democracy can be understood not only as a technological innovation, but also as a political opportunity structure that enables broader and more flexible citizen participation.

Theoretically, the relationship between digital democracy and political participation can be explained through the civic voluntarism and mobilization perspectives. The civic voluntarism model argues that participation is influenced by resources, political engagement, and recruitment networks. Digital democracy strengthens these three dimensions by providing citizens with political information, online communication skills, social networks, and invitations to participate. At the same time, digital platforms create mobilizing stimuli through campaign messages, peer influence, calls for collective action, and exposure to public issues. As a result, citizens who are active in digital democratic spaces are more likely to develop interest, efficacy, and motivation to participate in politics.

Empirical research supports this expectation. Boulianne (2020), in a meta-analysis of more than 300 studies across 50 countries, found that digital media use is generally associated with civic and political participation, and that this relationship has become stronger over time due to the spread of social networking sites and online participation tools. Saud, Ida, Mashud, Yousaf, and Ashfaq (2023), in a study of youth in Indonesia and Pakistan, found that social media functions as a platform for political communication, political expression, and digital democracy. Their findings show that social media political culture is strongly correlated with political efficacy, democracy, and civic engagement.

Evidence from Indonesia also strengthens this argument. Ida, Mashud, Saud, Yousaf, and Ashfaq (2025) found that social networks influence youth political participation by enabling young citizens to remain engaged in socio-political issues, voice opinions, and participate in political activities. Their study also emphasizes that online engagement should be connected with offline participation in order to create a more participatory and inclusive democracy. This indicates that digital democracy can extend conventional participation by creating pathways from online political communication to offline political involvement.

A systematic review by Congge, Guillamón, Nurmandi, Salahudin, and Sihidi (2023) also shows that digital democracy creates new spaces for public involvement in democratic processes. Digital platforms can help citizens access political information, engage in policy debates, monitor public institutions, and participate in decision-making. However, the effectiveness of digital democracy depends on institutional support, public knowledge, inclusiveness, and the quality of digital infrastructure. Similarly, Weinhardt, Fegert, Hinz, and van der Aalst (2024) argue that digital democratic systems must be designed with transparency, literacy, inclusion, accountability, and responsiveness in order to strengthen democratic resilience.

Thus, digital democracy is expected to increase political participation by lowering participation costs, improving access to political information, expanding communication networks, strengthening political efficacy, and creating new channels for civic engagement. Nevertheless, the effect of digital democracy on political participation is not automatic. It depends on whether digital platforms are inclusive, trustworthy, user-friendly, and connected to meaningful democratic processes. When digital democracy provides credible information and real opportunities for citizen involvement, it can encourage citizens to participate more actively in public affairs. Based on this theoretical and empirical explanation, this study proposes the following hypothesis:

Hypothesis 3 (H3). *Digital Democracy positively influences Political Participation.*

*2.5.4. Political Literacy (PL) as a Mediator*

Political literacy can be conceptualized as a set of integrated competencies that combine political knowledge, interpretive skills, critical awareness, and democratic values. These competencies enable citizens to understand political institutions, evaluate political information critically, recognize their rights and responsibilities, and identify effective channels for influencing public decision-making. Schmitt, Baake, and Kero (2024) explain that civic education in digitalized environments is expected to foster political knowledge, democratic attitudes, political self-efficacy, and willingness to participate. Similarly, Prasetiyo, Rahmawati, and Rahayu (2025) emphasize the importance of civic literacy in digital democratic learning, especially because citizens need the ability to understand, evaluate, and participate in democratic life.

In a digital democratic environment, political literacy is often developed through sustained interaction with online political content, social media discussions, civic technology platforms, online consultation mechanisms, and digital civic education initiatives. These digital spaces expose citizens to policy debates, competing viewpoints, electoral information, public criticism, and procedural knowledge about governance. Saud, Ida, Mashud, Yousaf, and Ashfaq (2023) show that digital spaces support political communication, political expression, democracy, and youth participation in virtual spheres. Weinhardt, Fegert, Hinz, and van der Aalst (2024) also argue that literacy is one of the essential pillars of resilient digital democracy, because citizens need the skills to navigate, criticize, and contribute to digital democratic spaces.

Previous studies suggest that digital democracy does not only provide channels for mobilization, but can also function as a learning environment that strengthens political literacy. Congge, Guillamón, Nurmandi, Salahudin, and Sihidi (2023) explain that digital democracy creates new spaces for community involvement in democratic life and that public knowledge is an important condition for quality digital democracy. Internews (2023) also warns that citizens who participate in digital political spaces without sufficient digital and political literacy may become more vulnerable to misinformation, disinformation, and manipulation. Therefore, literacy-building efforts are necessary to make digital participation more meaningful and democratic.

At the same time, political literacy is theoretically and empirically linked to political participation. Citizens with higher political literacy are more likely to understand political issues, feel capable of participating, evaluate political information critically, and use available democratic channels to express their interests. Revalina, Suryanef, Rafni, Putra, and Ockta (2024) found a significant positive relationship between political literacy and the political participation of first-time voters ahead of the 2024 election in Kerinci Regency. Noprianto, Wantu, and Djaafar (2025) also show that political education programs can improve the quality of voter participation by increasing citizens’ understanding of political rights, electoral procedures, and democratic responsibilities.

Taken together, these arguments suggest an indirect pathway from digital democracy to political participation through political literacy. Digital democracy provides accessible, interactive, and often youth-oriented spaces where citizens can learn about political systems, observe political debates, express preferences, and engage with public issues. These experiences can strengthen political literacy by increasing political knowledge, critical awareness, and internal political efficacy. In turn, politically literate citizens are more likely to transform digital exposure and democratic opportunities into concrete forms of political participation, such as voting, joining campaigns, participating in public discussions, contacting officials, or engaging in civic advocacy.

Thus, political literacy can be understood as a cognitive and psychological mechanism that explains how digital democracy influences political participation. Digital democracy acts as a contextual resource that provides information, interaction, and opportunities for civic learning, while political literacy converts these opportunities into informed and meaningful participatory behavior. Based on this theoretical and empirical explanation, this study proposes the following mediation hypothesis:

Hypothesis 4 (H4). *Digital Democracy positively influences Political Participation through Political Literacy as a mediator.*

*2.5.5. Post-Truth Politics (PT) as a Moderator*

Post-truth politics describes a political environment in which appeals to emotion, identity, and personal belief become more influential than verifiable facts in shaping public opinion. In such an environment, the shared standards of truth, rational debate, and institutional trust are weakened. Parker (2024) explains that the post-truth era challenges democratic norms and institutions because public reasoning is increasingly shaped by distrust, emotional narratives, and contested knowledge. Zembylas (2026) also argues that post-truth politics threatens the construction of a shared public world and highlights the importance of political education, judgment, plurality, and responsible participation in responding to post-truth challenges.

In digitally mediated political campaigns, post-truth dynamics are strengthened by the large-scale circulation of misinformation, disinformation, conspiracy narratives, hate speech, and emotionally charged political content. Vasist, Chatterjee, and Krishnan (2024) show that disinformation campaigns and hate speech play a significant role in polarizing societies. Ecker et al. (2022) further explain that misinformation is shaped by cognitive, social, and affective factors, and that false beliefs may continue to influence people even after corrections are provided. As a result, citizens are exposed to dense and contradictory political information streams and must continuously distinguish between accurate information, misinformation, and deliberate manipulation.

Within this context, political literacy can be understood as a set of knowledge, interpretive skills, critical awareness, and democratic values that enable citizens to understand political institutions, assess political messages, and connect public issues with their own rights and responsibilities. Septian and Wulandari (2024) show that political literacy is important for Generation Z participation in elections, especially because young citizens need the ability to understand political information and participate critically in democratic processes. Civic and media literacy studies also suggest that literacy can help citizens resist disinformation and engage more meaningfully in democracy. Lilja et al. (2024) argue that civic literacy can strengthen democratic resilience against disinformation, while Austin, Borah, and Domgaard (2021) found that media literacy is associated with political engagement and lower acceptance of disinformation. Muringa and Adjin-Tettey (2024) similarly found that media literacy enhances political knowledge, critical analysis of media messages, and democratic engagement among university students.

However, the influence of political literacy on political participation may depend on the broader information environment. Vozab (2023) shows that the relationship between news media literacy and political participation is not always uniform across generations and forms of participation. This indicates that literacy does not automatically lead to political action. In a highly post-truth environment, even politically literate citizens may face information overload, distrust, emotional fatigue, and uncertainty about which political actors or institutions deserve support. These conditions can reduce the extent to which political knowledge and critical skills are translated into concrete political participation.

Therefore, post-truth politics can be theorized as a moderating variable that weakens the positive relationship between political literacy and political participation. Under low post-truth conditions, citizens with higher political literacy are more likely to understand political issues, trust democratic procedures, and participate through voting, discussion, campaigning, or civic advocacy. However, under high post-truth conditions, the same literacy may not produce the same level of participation because citizens encounter manipulative narratives, polarized debates, distrust toward institutions, and uncertainty about the credibility of political information. In this situation, political literacy remains important, but its ability to motivate participation becomes weaker.

This does not mean that political literacy is irrelevant in post-truth politics. Rather, political literacy is necessary but may be insufficient when the public sphere is dominated by disinformation, identity-based propaganda, and affective polarization. Citizens with high political literacy may still be better able to identify manipulation, verify information, and avoid false claims. Nevertheless, post-truth politics can reduce their willingness to participate if the political environment appears too polarized, dishonest, or institutionally untrustworthy. In this sense, post-truth politics functions as a contextual barrier that limits the behavioral effect of political literacy.

Based on this theoretical and empirical explanation, this study proposes the following moderation hypothesis:

Hypothesis 5 (H5). *Post-Truth Politics is weakening the influence of Political Literacy toward Political Participation.*

The research model of this research is shown in Figure 1:

**Figure 1.**
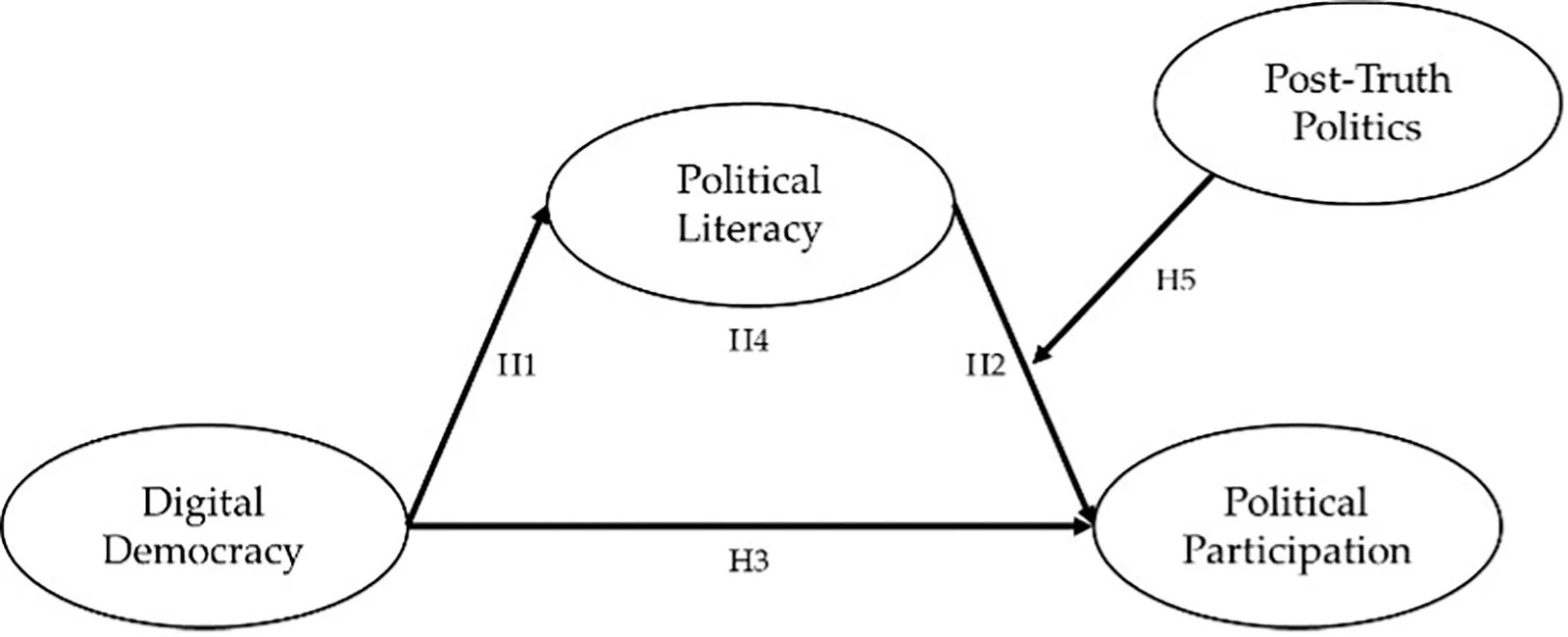
Research model. Source: Constructed by the authors (2025).

## Methodology

### 3.1. Research subjects and sampling techniques

This study employed a quantitative research design using a survey approach. The survey was conducted using a structured questionnaire to measure Digital Democracy, Political Literacy, Political Participation, and Post-Truth Politics. Each variable was measured using a four-point Likert scale to obtain standardized and measurable responses from participants. The study commenced on July 7, 2025, and participant enrolment was conducted from August to September 2025.

The population of this study consisted of Generation Z voters in Indonesia, totaling 50,645,669 individuals based on the 2024 Permanent Voter List. The sample included 534 respondents selected using proportionate stratified sampling across 34 provinces. This sampling technique was used to ensure that respondents were proportionally represented based on the distribution of Generation Z voters in each province.

### 3.2. Research instruments

The research instrument used in this study was a structured questionnaire. The questionnaire consisted of two main sections. The first section collected demographic data, including age, gender, education level, occupation, and social media use for accessing political information. The second section contained Likert-scale items measuring Digital Democracy, Political Literacy, Political Participation, and Post-Truth Politics.

The questionnaire was designed to measure respondents’ online political behavior, political attitudes, political awareness, and participation tendencies. Validity and reliability tests were conducted to ensure that the questionnaire items were appropriate, consistent, and able to measure the intended constructs.

### 3.3. Data analysis technique

Quantitative data were analyzed using Structural Equation Modeling (SEM) with AMOS. The analysis included descriptive statistics, validity and reliability tests, and Confirmatory Factor Analysis (CFA). The structural model tested direct, indirect (mediation), and moderating effects. Model fit was evaluated using indices such as Chi-square, RMSEA, GFI, CFI, and TLI. Bootstrapping was used to test indirect effects, while assumptions such as normality and model fit were also considered.

## Results

The findings of a survey conducted by researchers on Gen Z respondents in Indonesia regarding various variables such as digital democracy, political literacy, post-truth politics, and political participation in the 2024 elections can provide answers to various issues in this study. A total of 534 respondents acknowledged or recognized themselves as belonging to the Gen Z category in Indonesia. The data show that 100% of the respondents identified themselves as Gen Z, meaning that almost all respondents represented this age group. In a study focusing on Indonesia, the distribution of respondents was recorded as almost throughout Indonesia.

The largest number of respondents were in West Java, Central Java, and East Java. The even distribution of respondents during the survey had several objectives such as representing the population. By distributing the survey to representatives from each region, it was possible to directly represent the characteristics of each region and the identity of the community, which determined respondents’ answers.

Furthermore, even distribution can prevent bias given that uneven distribution may reflect regional preferences or conditions rather than representing the entire country. For example, in determining the impact of digital democracy, political literacy, and post-truth politics on Gen Z participation, if the survey is not distributed evenly, the results may be biased owing to the influence of regional backgrounds, cultures, and education. Furthermore, a survey that is distributed evenly can produce valid and reliable data so that it can be measured with consistent and stable reliability if the results are requested to be recalculated. The respondents’ gender results were divided into two categories: male and female. There were 317 males (59.36%) and 217 females (40.64%) with a total validity of 534.

Based on the results presented in Table 1, the four research constructs–Digital Democracy (DD), Political Literacy (LP), Post-Truth Politics (PT), and Political Participation (PP)–have Cronbach’s alpha values >0.7, and Composite Reliability (CR) > 0.7. This indicates that all constructs have good internal consistency and can therefore be declared reliable. The Average Variance Extracted (AVE) value for each construct is above 0.5, which means that the variance of the indicators can be explained more by the construct than by error. Thus, it can be concluded that the four constructs had adequate convergent validity.

**Table 1.** Validity and reliability tests.

Construction	Number of Indicators	CA	CR	AVE
DD	24	0.957	0.961	0.512
LP	11	0.932	0.942	0.599
PT	18	0.94	0.947	0.509
PP	23	0.962	0.965	0.546

Source: Processed by the authors based on research data (2025)

Based on the results of the SEM analysis using AMOS in Table 2 **,** the research model has a good level of feasibility (CMIN/DF = 2.14, GFI = 0.92, CFI = 0.95, TLI = 0.94, RMSEA = 0.056) so that the model can be used to test the hypotheses. The mediation test results show that political literacy mediates the influence of digital democracy on political participation (indirect effect = 0.29), thus supporting H4. This means that digital democracy not only has a direct influence but also strengthens political participation through increased political literacy. The moderation test results show that post-truth politics moderates the relationship between political literacy and political participation in a negative direction (moderation coefficient = −0.21; p-value = 0.006), thus accepting H5. This means that the higher the political literacy, the weaker is the influence of post-truth politics on Gen-Z’s political participation.

**Table 2.** Model goodness of fit index results.

Index	Cut-off Value	Result	Decision
Chi-square/df (CMIN/DF)	≤ 3,00	2,14	Fit
GFI	≥ 0,90	0,92	Fit
CFI	≥ 0,90	0,95	Fit
TLI	≥ 0,90	0,94	Fit
RMSEA	≤ 0,08	0,056	Fit

Source: Processed by the authors based on research data (2025)

Table 3 summarizes the SEM findings derived from the AMOS analysis, showing that all five hypotheses were accepted because the paths had positive and significant coefficients (p < 0.05). The mediating effect was proven to be significant through bootstrapping, while the moderating effect was also proven to be significant through moderation testing. Based on Table 3, all the hypotheses supported by significant coefficients, including a significant negative moderating effect, were confirmed.

**Table 3.** Hypothesis testing.

Hypotheses	Path	β Estimate	p-value	Decision
H1	DD → PL	0.61	0,000	Accepted
H2	LP → PP	0.48	0,000	Accepted
H3	DD → PP	0.37	0,000	Accepted
H4	DD → LP → PP	0.29	0.002	Accepted
H5	LP × PT → PP	−0.21	0.006	Accepted

Source: Processed by the authors based on research data (2025)

## Discussion

Studies on Generation Z in Indonesia have grown alongside the rapid expansion of internet and social media use. Known for their creativity, expressiveness, and strong attachment to digital platforms, Indonesian Gen Z is closely associated with social media culture. While millennials were highlighted in the 2019 election, Generation Z gained greater attention in 2024 as one of the largest groups of new voters and as a key driver of political awareness through digital platforms.

Generation Z differs from previous generations in its critical outlook. Unlike millennials, who may have become disillusioned over time, Gen Z tends to question systems from the outset. Growing up with technology and unlimited access to information, they are often referred to as the “internet generation.”

They also prefer interactive digital features such as polls, live streaming, and real-time comments to communicate and engage with others. This reflects their evolving interaction patterns shaped by technological and cultural changes.

### 5.1. The development of digital democracy affects Gen Z’s political literacy in the 2024 elections

Digital democracy is expected to promote citizens’ independence and rationality by providing wider access to political information, public discussion, and political participation through digital platforms. The emergence of social media has created new spaces for citizens to communicate, express aspirations, criticize government policies, and debate national and international political issues. Before the rapid development of social media, public debate was more limited by distance, time, and geographical barriers. Therefore, digital democracy can reduce participation barriers and make political communication more open, accessible, and interactive. Digitalization also has the potential to increase public participation by improving access to information, strengthening political inclusion, and making civic engagement more practical ( OECD, 2024).

Digital democracy can also strengthen public monitoring of government performance. Through digital platforms, citizens can observe public policies, respond to government programs, and express criticism more directly. In this sense, digital democracy does not only function as a communication channel, but also as a mechanism for public accountability. Digital resources for political action allow citizens and civic technology organizations to challenge, reform, and improve democratic processes ( Berg & Hofmann, 2021). Similarly, Congge et al. (2023) explain that digital democracy provides a new space for community involvement in democratic life.

The relationship between digital democracy and political literacy is mutually reinforcing. Political literacy enables citizens to participate more actively, critically, and responsibly in digital democratic spaces. Citizens with stronger political literacy are more capable of evaluating political information, understanding institutional mechanisms, identifying misinformation, and making informed political decisions. At the same time, digital democracy serves as a platform that can strengthen political literacy by exposing citizens to political news, policy debates, public criticism, campaign information, and civic education content. Thus, digital democracy can become both a medium of participation and a tool for political learning.

In the 2024 Indonesian election, Generation Z became an important group of voters. The General Election Commission reported that the total number of voters in the 2024 election was 204,807,222, with around 46.8 million Gen Z voters and 66.8 million millennial voters. This shows that young voters had a significant position in the 2024 electoral landscape. However, the political importance of Gen Z should not only be understood from their number as voters, but also from their active presence in digital spaces. Gen Z tends to use social media to access political information, follow public debates, give criticism, and respond to government policies. Therefore, their digital behavior can contribute to public monitoring and democratic participation.

Social media has become an important part of contemporary electoral politics. Gibson (2015) argues that digital media have introduced a new form of citizen-initiated campaigning, in which citizens and grassroots actors can participate in campaign-related activities outside traditional party structures. This means that political campaigns are no longer controlled only by parties and candidates, but are also shaped by citizens’ online discussions, content sharing, and digital mobilization. In the context of Generation Z, social media can function as a space where political information is accessed, debated, and circulated.

However, the development of digital democracy and political literacy has not yet reached an ideal pattern. Digital platforms can support political education and participation, but they can also spread misinformation, disinformation, hate speech, and manipulative political content. Guess and Munger (2023) note that digital literacy is still a concept with limited consensus in terms of definition and measurement, while also showing that individuals have different levels of ability in finding and evaluating online information. This indicates that the benefits of digital democracy depend on citizens’ capacity to process political information critically.

Therefore, improving political literacy in digital democracy requires not only access to technology, but also ethical, educational, and regulatory support. Christodoulou and Iordanou (2021) emphasize that digital media and technology should reflect broader ethical considerations and should not be treated as if the digital world were separated from human subjects and democratic values. Ethical awareness in digital media is important because political information circulating online can influence citizens’ perceptions, decisions, and participation.

In this context, digital democracy is expected to influence the political literacy of Generation Z in Indonesia. The more social media and digital platforms develop as spaces for political communication, the more frequently Gen Z encounters political information, campaign messages, policy debates, and public criticism. If supported by adequate political literacy and digital literacy, this exposure can help Gen Z become more critical, informed, and participatory citizens. However, without sufficient literacy, the same digital environment may also make them vulnerable to misinformation, polarization, and political manipulation.

### 5.2. Advances in political literacy and their effect on Gen Z Political Participation in Indonesia

Improving political literacy is essential for strengthening Generation Z’s political participation in Indonesia. Political literacy is not merely the ability to recognize political information, but also the capacity to understand political institutions, evaluate public issues critically, and participate responsibly in democratic processes. Dudley and Gitelson (2002) argue that political literacy is closely related to civic education and civic engagement because political knowledge helps citizens understand how and why they should participate in public life.

In the context of the 2024 Indonesian election, political literacy became increasingly important because Generation Z represented a significant group of voters. Their participation cannot be measured only by voting behavior, but also by their ability to evaluate candidates’ programs, criticize public policies, respond to political issues, and express opinions through digital platforms. For Gen Z, political participation often occurs through social media, where they access political information, discuss public issues, and communicate their political views.

Political literacy can encourage both electoral and non-electoral participation. Gen Z voters with stronger political literacy are more likely to understand electoral procedures, evaluate political promises, identify misinformation, and participate in political discussion. Yanto and Sari (2025) found that social media use was significantly correlated with political literacy and democratic participation among Indonesian Gen Z students. Their study also showed that although most respondents relied on social media as their main source of political information, many still had limited ability to verify the accuracy of that information.

Therefore, strengthening political literacy among Gen Z requires educational strategies that are engaging, practical, and suitable for their digital habits. Political education should not be delivered only through formal, rigid, or ceremonial activities, but also through interactive digital content, issue-based discussions, short videos, infographics, and social media campaigns. Borneta et al. (2025) show that political education content on Instagram can increase Gen Z’s political awareness and understanding when the content is accessible, visually clear, concise, and relevant.

Political literacy also involves knowledge, skills, and attitudes. Knowledge helps Gen Z understand political systems, electoral processes, policy-making, and democratic values. Skills enable them to evaluate political information, verify sources, communicate arguments, and participate in digital discussions. Attitudes shape their political choices, sense of responsibility, tolerance, and willingness to contribute to democratic life. Weiss (2020) emphasizes that youth political participation is multidimensional and should be understood through both conventional and non-conventional forms of engagement.

However, the development of Gen Z political participation also faces challenges. Social media can support political education, but it can also expose young voters to hoaxes, emotional narratives, political polarization, and manipulative campaign messages. Septian and Wulandari (2024) note that hoaxes and money politics remain challenges for novice voters in the 2024 election. For this reason, political literacy must strengthen not only political knowledge, but also critical thinking and ethical digital behavior.

Overall, political literacy has an important effect on Gen Z political participation in Indonesia. When Gen Z voters are politically literate, they are more capable of responding to political issues rationally, evaluating political information critically, and participating actively through voting, discussion, advocacy, and social media engagement. Thus, strengthening political literacy is necessary to ensure that Gen Z’s digital participation contributes positively to democratic development in Indonesia.

### 5.3. Digital democracy influences Gen Z political participation in the 2024 elections in Indonesia

The development of digital democracy has influenced Generation Z’s political participation in the 2024 Indonesian election. Digital democracy enables citizens to access political information, express aspirations, criticize government policies, and engage in public debate through digital platforms. In this context, social media, online discussion forums, e-government services, and civic technology platforms provide new spaces for citizens to participate in democratic processes. Van Dijk (2012) explains that digital democracy involves the use of digital media in political communication, including information provision, public discussion, and decision-making processes.

As a generation that has grown up in the digital era, Gen Z tends to express political participation through social media and other online platforms. Their participation is not limited to voting, but also includes sharing political content, commenting on public issues, criticizing political actors, joining online campaigns, and responding to government policies. This shows that digital democracy can expand conventional participation by creating more flexible and accessible channels for political engagement. Boulianne (2020) found that digital media use is generally associated with civic and political participation, while Saud et al. (2023) show that digital spaces support youth political communication, civic engagement, and democratic participation.

In the 2024 election, Generation Z became an important voter group in Indonesia. According to KPU data, the total number of voters in the 2024 election was 204,807,222. Of this number, Generation Z voters reached 46,800,161 people, or 22.85% of the total electorate, while millennial voters reached 66,822,389 people, or 33.60%. Together, Gen Z and millennials accounted for 56.45% of voters. This demographic composition encouraged political parties, candidates, and election organizers to use digital communication strategies that were more attractive to young voters.

The influence of digital democracy can also be seen in the way political actors adapted their campaign strategies. Political messages were increasingly packaged through short videos, social media posts, memes, informal language, visual content, and interactive communication. This strategy reflects the effort to reach young voters who are familiar with digital culture. Gibson (2015) argues that social media has contributed to the rise of citizen-initiated campaigning, in which political communication is no longer dominated only by parties and candidates, but also shaped by citizens’ online activities.

However, the effect of digital democracy on Gen Z political participation is not always simple. The survey results of this study show that many respondents did not choose extreme answers, such as strongly agree or strongly disagree, but tended to position themselves in moderate response categories. This may indicate that Gen Z voters are not always fully confident or consistent in evaluating digital democracy and political participation. Their responses may reflect critical attitudes, selective trust, or uncertainty toward political information circulating online.

Digital democracy also creates challenges. Although digital platforms make participation easier, they also increase exposure to misinformation, disinformation, hate speech, and political polarization. Asimakopoulos et al. (2025) explain that digital technologies can support democratic participation, but they also create risks related to misinformation, privacy, digital inequality, and manipulation. Therefore, digital democracy requires not only access to technology, but also political literacy, digital literacy, ethical communication, and institutional transparency.

An important example of digital democracy in Indonesia can be seen in the public response to the 2024 Regional Election Law revision controversy. Online movements such as #KawalPutusanMK and #PeringatanDarurat showed how social media could mobilize public criticism and encourage offline collective action. This indicates that digital platforms can function as spaces for democratic monitoring, especially when citizens perceive a threat to democratic procedures.

Overall, digital democracy has an important influence on Gen Z political participation in Indonesia. It provides wider access to political information, creates interactive spaces for discussion, and lowers barriers to participation. However, its democratic value depends on whether young citizens are able to process political information critically and whether digital platforms are used to strengthen, rather than weaken, democratic participation. Therefore, digital democracy can increase Gen Z political participation when it is supported by political literacy, reliable information, and responsible digital engagement.

### 5.4. Political literacy can mediate the influence of the relationship between digital democracy and political participation in the 2024 elections

Political literacy plays an important mediating role in the relationship between digital democracy and political participation. In the digital era, citizens are exposed to a large amount of political information through social media, online news, digital campaigns, and civic technology platforms. However, access to information alone does not automatically lead to meaningful political participation. Citizens need political literacy in order to understand, evaluate, and use political information critically.

Digital democracy provides opportunities for Generation Z to access political information, express opinions, follow campaign debates, and participate in public discussions. These opportunities can strengthen political literacy when Gen Z voters are able to process information rationally, compare different perspectives, identify misinformation, and understand political issues. In this sense, political literacy becomes the bridge between digital exposure and political action.

Previous studies support this argument. Erdem et al. (2023) show that digital literacy mediates the relationship between media literacy and digital citizenship, indicating that literacy-related competencies are important in connecting media exposure with civic behavior. Lee, Cho, and Kim (2023) also explain that media literacy education and parental mediation can increase youth political participation through surveillance motivation, reflection, and online news engagement. This suggests that the ability to process political information critically can encourage young citizens to become more engaged in political life.

In the context of the 2024 Indonesian election, political literacy helped Generation Z transform digital democratic experiences into political participation. Gen Z voters did not only receive political information through social media, but also evaluated candidate messages, responded to campaign issues, joined political discussions, and expressed opinions online. When supported by adequate political literacy, these digital activities could encourage more informed participation, including voting, campaign engagement, policy criticism, and civic discussion.

Political literacy also protects Gen Z voters from the negative effects of digital democracy. Without sufficient literacy, digital platforms may expose young voters to misinformation, disinformation, emotional narratives, and manipulative political content. This can create confusion, distrust, or passive political behavior. By contrast, politically literate citizens are more capable of verifying information, distinguishing facts from hoaxes, understanding different viewpoints, and participating in democracy more responsibly.

Therefore, the influence of digital democracy on political participation depends partly on the level of political literacy. Digital democracy provides access, interaction, and opportunities for participation, while political literacy determines whether these opportunities can be converted into informed and meaningful political behavior. In this study, political literacy functions as a cognitive and psychological mechanism that explains how digital democracy influences Gen Z political participation in the 2024 Indonesian election.

Overall, political literacy strengthens the positive role of digital democracy by helping Gen Z voters understand political information, evaluate public issues critically, and participate more consciously in democratic processes. Thus, political literacy is not only an outcome of digital democracy, but also a key mediator that connects digital democratic practices with political participation.

### 5.5. Post-truth politics can moderate the relationship between Gen Z’s political literacy and political participation in the 2024 elections

The rapid growth of social media and digital communication has contributed to the emergence of post-truth politics. Post-truth politics refers to a condition in which objective facts become less influential in shaping public opinion than appeals to emotion, identity, and personal belief. In this environment, political information is often circulated quickly without adequate verification, allowing misinformation, disinformation, hoaxes, and emotionally charged narratives to spread widely.

In the context of digital politics, post-truth politics can weaken the relationship between political literacy and political participation. Political literacy should help citizens understand political institutions, evaluate political information, and participate critically in democratic processes. However, when the public sphere is dominated by post-truth dynamics, political literacy may not always be translated into active participation. Citizens may experience confusion, distrust, fatigue, or uncertainty because they are exposed to contradictory political information and manipulative narratives.

Social media intensifies this condition because its algorithmic systems often expose users to content that aligns with their preferences. This can create echo chambers or filter bubbles, where individuals repeatedly encounter similar political views and become less exposed to alternative perspectives. Bozdag and van den Hoven (2015) explain that filter bubbles can threaten democratic dialogue because users may be separated from information that challenges their existing beliefs. In this situation, political participation may become more emotional, polarized, and identity-based rather than rational and deliberative.

Post-truth politics is also closely related to misinformation and disinformation. Waisbord (2018) argues that fake news reflects the unstable condition of contemporary public communication and the contested role of news in shaping belief. Ecker et al. (2022) further explain that misinformation belief is influenced by cognitive, social, and affective factors, and that false information may continue to affect people even after correction. This is particularly important for Generation Z, who rely heavily on digital platforms for political information and public discussion.

In the 2024 Indonesian election, Generation Z became an important voter group and was highly active in digital spaces. Their political participation was shaped not only by access to political information, but also by their ability to evaluate the credibility of that information. Septian and Wulandari (2024) show that hoaxes and money politics remained challenges for Generation Z voters in the 2024 election. Similarly, Yanto and Sari (2025) found that social media plays an important role in shaping political literacy and democratic participation among Gen Z in the post-truth era, although the spread of disinformation remains a major challenge.

Therefore, political literacy is necessary but not always sufficient in a post-truth environment. Gen Z voters with strong political literacy may be better able to verify information, identify hoaxes, and evaluate political claims critically. However, when post-truth politics is high, the positive effect of political literacy on participation may become weaker because digital spaces are filled with emotional appeals, distrust, polarization, and manipulative content. As a result, even politically literate citizens may become less willing to participate if they perceive politics as dishonest, confusing, or overly conflictual.

This means that post-truth politics acts as a contextual barrier that reduces the behavioral effect of political literacy. Under low post-truth conditions, political literacy is more likely to encourage political participation because citizens can access more reliable information and engage in healthier public debate. Under high post-truth conditions, however, political literacy may have a weaker effect because the information environment is less trustworthy and more emotionally polarized.

Overall, post-truth politics moderates the relationship between political literacy and political participation among Generation Z in Indonesia. Strengthening political literacy remains important, but it must be accompanied by media literacy, fact-checking habits, ethical digital communication, and institutional efforts to reduce disinformation. Without these supports, post-truth politics may weaken the ability of political literacy to encourage meaningful political participation.

## Conclusions

This study concludes that digital democracy significantly strengthens Generation Z’s political literacy in the 2024 Indonesian election. Social media and other digital platforms provide spaces for political communication, public discussion, expression of aspirations, and access to political information, which help Gen Z develop greater political awareness.

Political literacy also has a significant influence on Gen Z’s political participation. Higher political literacy enables Gen Z voters to analyze political information critically, avoid misinformation and disinformation, and participate more actively through voting, online discussion, campaign engagement, and public expression.

Furthermore, political literacy mediates the relationship between digital democracy and political participation. Digital democracy provides access to information and interaction, while political literacy helps transform these digital experiences into meaningful political action. In this sense, political literacy functions as a bridge between digital democratic practices and Gen Z’s participation in the 2024 election.

Post-truth politics moderates the relationship between political literacy and political participation by potentially weakening the positive influence of political literacy. Hoaxes, fake news, misinformation, and disinformation can distort political understanding and reduce the quality of participation. However, the findings indicate that political literacy still plays a stronger role in encouraging Gen Z’s political participation than the weakening effect of post-truth politics.

Theoretically, this study contributes to the development of digital democracy and political literacy studies. Digital democracy should not only be understood as a tool for expanding participation, but also as a new political ecosystem that shapes how citizens access information, express opinions, and participate in democratic processes. Political literacy should also be understood as a multidimensional capacity that includes knowledge, critical thinking, democratic values, and the ability to distinguish facts from manipulation in digital political spaces.

Practically, this study provides insights for political parties, election organizers, government institutions, and civil society organizations in preparing more effective strategies to increase young voters’ participation in future elections, especially the 2029 election. Generation Z should be seen not only as voters, but also as important political actors whose digital habits, creativity, and critical awareness can contribute to the strengthening of democracy and Indonesia’s long-term development toward Golden Indonesia 2045.

This study has several limitations. It focuses on Generation Z voters in the context of the 2024 Indonesian election and uses a quantitative approach, so the findings may not fully capture deeper personal experiences or changes across time. Future research is recommended to use longitudinal designs, examine other moderating variables such as social media algorithms, compare different generations or countries, and integrate experimental or big data analysis to understand the influence of digital political content more precisely.

## Ethical approval

This study involved human participants and was conducted in accordance with the Declaration of Helsinki and the ethical guidelines for research involving human respondents at Universitas Airlangga. Ethical approval was obtained from the Graduate School of Universitas Airlangga, approved by the Vice Director I, Prof. Dr. Rudi Purwono, S.E., M.SE, under approval number 2212/B/UN3.SPS/I/PT.01.05/2025, dated 7 July 2025. All research procedures were designed to protect participants’ rights, confidentiality, anonymity, and voluntary participation.

## Informed consent

Informed consent was obtained from participants before data collection. Participants were informed about the purpose of the study, the voluntary nature of their participation, the confidentiality of their responses, and their right to withdraw from the study. For the expert panel involved in the research process, written informed consent was obtained. For the questionnaire survey, participants provided consent before completing the questionnaire. No personally identifiable images were collected in this study, and all responses were treated confidentially and used only for research purposes.

## Data Availability

The underlying data supporting the findings of this study are available on Figshare under the title “Underlying_Data_Anonymized.xlsx” with the following DOI: https://doi.org/10.6084/m9.figshare.32833550.v1 ( Latuconsina, 2026b). The dataset includes anonymized survey responses, variable descriptions, and data used for the statistical analyses reported in this article. All personal identifiers were removed before public sharing. Data is available under the terms of the Creative Commons Attribution 4.0 International license. The extended data for this study are available on Figshare under the title “Questionnaire Instrument for the Study on Digital Democracy, Political Literacy, Post-Truth Politics, and Generation Z Political Participation in the 2024 Indonesian Election” with the following DOI: https://doi.org/10.6084/m9.figshare.32833664.v1 ( Latuconsina, 2026a). The extended data include the questionnaire instrument used to measure Digital Democracy, Political Literacy, Post-Truth Politics, and Political Participation among Generation Z voters in the 2024 Indonesian election. Data is available under the terms of the Creative Commons Attribution 4.0 International license.
