# Digital Democracy and Gen Z Political Participation in Indonesia's 2024 Election: The Mediating Role of Political Literacy and the Moderating Role of Post-Truth Politics

**DOI:** 10.12688/f1000research.185707.2

**Published:** 2026-10-17

**Authors:** Agust Jovan Latuconsina, Fendy Suhariadi, Suparto Wijoyo, Ian Firstian Aldhi

**Affiliations:** 1 Universitas Airlangga, Surabaya, East Java, Indonesia

**Keywords:** Digital Democracy; Political Literacy; Post-truth Politics; Political Participation; Gen Z

## Abstract

**Background:**

Digital democracy has expanded the ways young citizens access political information and participate in public life, while post-truth politics may complicate how political knowledge is translated into participation. This study examines the relationships among Digital Democracy, Political Literacy, Post-Truth Politics, and Political Participation among Generation Z in the context of the 2024 Indonesian election.

**Methods:**

A quantitative cross-sectional survey using non-probability purposive sampling included 500 Generation Z voters aged 18–28 years who were registered in the 2024 Permanent Voter List and resided across all 38 provinces of Indonesia. Data were collected in August–September 2025 using a four-point Likert-scale questionnaire; items referring to the 2024 election were therefore retrospective self-reports. Direct, mediating, and moderating relationships were tested using covariance-based Structural Equation Modeling with AMOS.

**Results:**

Digital Democracy was positively associated with Political Literacy (β = 0.491, p < 0.001) and Political Participation (β = 0.296, p < 0.001), while Political Literacy was positively associated with Political Participation (β = 0.390, p < 0.001). Political Literacy partially mediated the Digital Democracy–Political Participation relationship (indirect effect = 0.192, 95% CI [0.144, 0.244]). Post-Truth Politics negatively moderated the Political Literacy–Political Participation relationship (β = −0.173, p < 0.001); simple slopes remained positive but decreased from 0.709 at low Post-Truth Politics to 0.362 at high Post-Truth Politics.

**Conclusions:**

The findings identify Political Literacy as a partial explanatory mechanism linking Digital Democracy with Political Participation and Post-Truth Politics as a contextual boundary condition under which the Political Literacy–Political Participation association is weaker. Because the study is cross-sectional and retrospective, the findings should be interpreted as statistical associations rather than causal effects.

## 1. Introduction

Digital platforms have become central to political communication and civic engagement, particularly for younger citizens who routinely encounter political information through social media and other online spaces. In Indonesia, this context is especially salient: internet penetration reached 79.5% in 2024, while Generation Z represented the largest age-group share among internet users ( Kementerian Komunikasi dan Digital, 2024). Digital media can widen access to information, public discussion, and political expression, providing additional channels for citizens to engage with public issues ( Saud et al., 2023; Weinhardt et al., 2024).

Recent scholarship increasingly treats digital democracy as more than the simple migration of political communication to online platforms. It involves changes in how citizens obtain information, deliberate, interact with institutions, and move between online expression and other forms of civic action. Berg and Hofmann (2021) emphasize that digital democracy is shaped by both technological infrastructures and institutional arrangements, while Asimakopoulos et al. (2025) show that information and communication technologies can broaden opportunities for citizen participation while also raising concerns about privacy, security, and unequal access. These tensions are important because the democratic value of digitalization depends not only on connectivity, but also on whether digital environments support meaningful participation and informed public reasoning ( Berg & Hofmann, 2021; Asimakopoulos et al., 2025; Christodoulou & Iordanou, 2021).

However, the expansion of digital participation does not guarantee informed or deliberative participation. Digital spaces also expose citizens to misinformation, disinformation, emotionally charged narratives, and selective information environments. Post-truth politics is relevant in this setting because public judgments may be shaped less by verifiable facts than by emotion and personal belief ( Oxford Languages, 2016). Research on misinformation and political disinformation further shows that information quality and affective dynamics can complicate how citizens interpret political messages and engage in public life ( Ecker et al., 2022; Vasist et al., 2024).

The problem is therefore not only exposure to false information, but the wider transformation of the information environment in which citizens make political judgments. Post-truth scholarship highlights how emotionally resonant claims, fragmented authority, and contested standards of evidence can alter political communication ( Corner, 2017; Giusti & Piras, 2021). In Indonesia, analyses of the digital public sphere similarly emphasize the coexistence of participatory opportunities and information disorder ( Utomo, 2022). This makes it necessary to examine not only whether political literacy is positively associated with participation, but also whether that association remains equally strong when respondents perceive the surrounding political-information environment as more post-truth oriented.

These tensions are particularly important for Generation Z, a cohort whose social and political interactions are deeply embedded in digital media. Their familiarity with digital platforms creates substantial opportunities for political learning and participation, but high exposure to rapidly circulating information also makes the ability to interpret and evaluate political content consequential. Political literacy therefore offers a plausible cognitive mechanism linking digital democratic experiences with political participation. Prior work shows that digital media use is generally associated with civic and political engagement ( Boulianne, 2020), while social media and digital democratic spaces can support political communication, public involvement, and political learning ( Congge et al., 2023; Saud et al., 2023; Weinhardt et al., 2024).

The Indonesian context further strengthens the relevance of this problem. Studies focusing on young voters suggest that social media can function simultaneously as an arena for political information, civic learning, campaign communication, and public expression. Evidence from the Indonesian Electoral Commission’s social-media environment indicates that platform-based communication can support political literacy among Generation Z ( Borneta et al., 2025), while other recent studies connect civic education and social-media engagement with young people’s political participation ( Septian & Wulandari, 2024; Suhariyanto & Rozak, 2025). Research on influencers and digital political communication also shows that the pathways into youth participation are increasingly embedded in online communication networks rather than confined to conventional campaign channels ( Venus et al., 2025).

Political literacy is more than factual political knowledge; it includes the capacity to understand institutions and issues, evaluate political information, and recognize meaningful channels for participation. Studies of young and first-time voters suggest that higher political literacy is associated with greater political participation ( Revalina et al., 2024), and digital civic education can strengthen political knowledge, efficacy, and willingness to participate ( Schmitt et al., 2024). Yet the existing literature does not fully explain whether political literacy serves as the mechanism through which digital democracy is associated with participation, rather than merely constituting another direct predictor. This distinction is theoretically important because access to digital political spaces and the capacity to translate that access into informed political action are not equivalent.

A second unresolved issue concerns the information environment in which political literacy operates. Literacy may not translate into political participation with equal strength under all conditions. Misinformation can persist even after correction ( Ecker et al., 2022), and the relationship between media literacy and participation varies across generations and forms of engagement ( Vozab, 2023). These findings suggest that post-truth politics may function as a boundary condition: when political discourse is more strongly characterized by emotionally charged, misleading, or contested information, the positive association between political literacy and political participation may be weaker. This possibility is especially relevant in digitally mediated elections, where information abundance coexists with challenges to information credibility.

Accordingly, the central gap addressed in this study is not simply whether digital democracy, political literacy, post-truth politics, and political participation can be combined in one model. Rather, the study addresses two theoretically linked questions: how digital democracy is associated with political participation through political literacy, and when the association between political literacy and participation becomes weaker under post-truth conditions. By treating political literacy as an explanatory mechanism and post-truth politics as a boundary condition, the study shifts attention from isolated direct relationships toward a conditional process perspective.

This study therefore examines Generation Z voters in the context of the 2024 Indonesian election. It tests the associations of Digital Democracy with Political Literacy and Political Participation, the association of Political Literacy with Political Participation, the mediating role of Political Literacy in the Digital Democracy-Political Participation relationship, and the moderating role of Post-Truth Politics in the Political Literacy-Political Participation relationship. Given the cross-sectional and retrospective survey design, these relationships are interpreted as statistical associations rather than causal effects.

## 2. Theoretical framework and hypothesis

### 2.1 Digital democracy

Digital democracy refers to the use of digital technologies to support political information, communication, deliberation, and participation across online and offline settings ( van Dijk, 2012; Lindner & Aichholzer, 2019). Its democratic value does not follow automatically from technological access: digital spaces can widen opportunities for expression and engagement while also reproducing inequalities, low-quality deliberation, and information problems ( Dahlgren, 2005; OECD, 2024).

Accordingly, this study treats digital democracy as an informational and participatory environment rather than as an inherently beneficial outcome. For Generation Z, this environment can provide frequent exposure to political news, debate, and interaction, but the usefulness of those opportunities depends partly on citizens’ capacity to interpret and evaluate political information. Recent work therefore places literacy, inclusion, transparency, and accountability among the conditions required for resilient digital democracy ( Weinhardt et al., 2024).

This conceptualization is consistent with the view of the Internet as a political space whose democratic consequences depend on the quality of communication and the architecture of participation. Dahlgren (2013) argues that digital media can create alternative spaces for political engagement, but those spaces do not automatically produce deliberation or civic equality. Platform design can also structure what information users encounter. The filter-bubble literature, for example, shows why personalization and selective exposure may narrow encounters with opposing viewpoints ( Dahlgren, 2013; Bozdag & van den Hoven, 2015). Thus, digital democracy is theoretically relevant to both political learning and participation, while its outcomes remain contingent on how citizens process the information to which they are exposed.

### 2.2 Political literacy

Political literacy encompasses knowledge of political institutions and processes together with the capacity to interpret political issues, evaluate competing claims, and understand one’s rights and responsibilities in democratic life ( Denver & Hands, 1990; Crick, 2000). It therefore extends beyond factual knowledge to include critical judgment and the practical ability to make informed political choices.

Political literacy is especially relevant in a high-information digital environment because it can reduce cognitive barriers to participation and strengthen citizens’ ability to connect political information with meaningful forms of engagement. Politically informed citizens are generally better positioned to recognize their interests and available channels of participation ( Delli Carpini & Keeter, 1996), while studies of digital and novice-voter contexts emphasize the need to evaluate online political information critically ( Ohme, 2019; Kurniasih et al., 2024). In the present model, political literacy is therefore positioned not merely as another correlate of participation, but as the explanatory mechanism linking digital democratic exposure with political participation.

Political-literacy research also suggests that knowledge alone is an incomplete representation of citizens’ democratic competence. Cassel and Lo (1997) conceptualize political literacy through cognitive, socialization, and structural explanations, whereas Eriksen (2020) emphasizes the capacity to understand and assess political claims and processes. Civic-education scholarship further links political literacy with the development of informed and reflective participation rather than simple exposure to political content ( Cassel & Lo, 1997; Eriksen, 2020; Dudley & Gitelson, 2002). These perspectives support treating Political Literacy as a mechanism that can help convert the opportunities provided by digital democratic spaces into more comprehensible and actionable political choices.

### 2.3 Post-truth politics

Post-truth politics describes a broader political-information environment in which objective facts may become less influential than emotion, identity, and personal belief in shaping public opinion ( Oxford Languages, 2016; Rietdijk, 2024). It should not be reduced to a single false claim. Misinformation may be inaccurate regardless of intent, disinformation involves strategic deception, fake news presents fabricated or misleading material in news-like form, and propaganda uses selective framing, repetition, and affective appeals to shape public opinion. These communicative forms can contribute to a post-truth environment when factual verification competes with emotionally resonant and identity-consistent narratives ( Corner, 2017; Giusti & Piras, 2021; Ecker et al., 2022).

A central mechanism in this environment is selective exposure and confirmation bias. Platform personalization and users’ own choices can narrow encounters with opposing viewpoints ( Bozdag & van den Hoven, 2015). When citizens preferentially accept information that fits prior beliefs, campaign claims about candidates, manifestos, leadership, and electoral issues may be trusted or shared because they confirm existing identities rather than because competing evidence has been critically interrogated. Repetition, source cues, and emotional appeals may further blur the distinction between verified and misleading content, while distrust of institutional or mainstream information sources can weaken shared standards of credibility ( Ecker et al., 2022; Rietdijk, 2024).

This study therefore conceptualizes Post-Truth Politics primarily as a contextual boundary condition. The issue is not simply whether post-truth perceptions are directly associated with participation, but whether they change the extent to which Political Literacy is translated into participation. Under stronger post-truth conditions, contradictory claims, credibility uncertainty, selective exposure, and affective narratives may make informed political judgment harder to convert into action. In this sense, post-truth is treated as a feature of a fragmented information ecology rather than simply as a synonym for fake news, while misinformation, disinformation, and propaganda-like communication are possible mechanisms through which that ecology is intensified. Trust, confirmation bias, and propaganda were not separately measured in the present survey and are therefore treated as theoretically plausible mechanisms rather than tested effects ( Vozab, 2023; Vasist et al., 2024; Lilja et al., 2024).

### 2.4 Political participation

Political participation refers to citizens’ voluntary activities intended to influence political selection, public decisions, or policy processes ( Huntington & Nelson, 1976; Leach et al., 2006). In contemporary democracies, participation includes both conventional activities such as voting and campaigning and digitally mediated activities such as political discussion, information sharing, advocacy, and other forms of online engagement ( Norris, 2002).

The civic voluntarism perspective suggests that participation is shaped by resources, political engagement, and recruitment networks ( Brady et al., 1995). Applied to this study, digital democracy can expand informational and interaction opportunities, political literacy can provide the cognitive capacity needed to use those opportunities, and post-truth politics can make the information environment less conducive to translating literacy into participation. This logic provides the integrative basis for the proposed direct, mediating, and moderating relationships.

### 2.5 Generation Z

Generation Z has developed within a social environment in which internet access, social media, and mobile communication are embedded in everyday interaction ( Singh & Dangmei, 2016). Although generational boundaries vary across sources, people born from the late 1990s onward are commonly treated as part of this cohort ( Dimock, 2019). Their political experiences are therefore closely intertwined with digital information environments, making this group particularly relevant for examining digital democracy, political literacy, and post-truth politics together.

At the same time, Generation Z should not be treated as politically or behaviorally homogeneous. Their familiarity with digital technologies may facilitate access to political information and public expression, but it can also increase exposure to information overload, low-quality content, and rapid-response communication. The present study therefore focuses on the relationships among the four constructs rather than assuming that digital nativeness itself produces a particular form or level of political participation.

Youth political participation is also multidimensional. Weiss (2020) notes that younger citizens may combine conventional activities such as voting with expressive, issue-based, and digitally mediated forms of participation. This helps explain why political literacy is relevant beyond electoral knowledge alone. Citizenship education can strengthen the skills and orientations through which young people recognize opportunities for civic action, although levels and forms of participation remain uneven across individuals and contexts ( Weiss, 2020; Pontes et al., 2019). The present model therefore treats political participation as a broad behavioral outcome while avoiding the assumption that all members of Generation Z respond identically to digital political environments.

### 2.6 Hypothesis development

The proposed model distinguishes between an explanatory mechanism and a contextual boundary condition. Digital democracy is expected to be positively associated with political participation both directly and indirectly through political literacy. Political literacy represents the cognitive mechanism through which access to and engagement with digital political environments can be connected to participation, whereas post-truth politics is expected to condition the strength of the political literacy–participation relationship.

*2.6.1 Digital Democracy (DD) and Political Literacy (PL)*

Digital democratic environments provide repeated opportunities to encounter political news, policy debates, civic information, and competing viewpoints. These opportunities can be associated with greater political understanding when users actively process and evaluate the information they encounter. Research has linked digital democratic spaces and social media use with political communication, civic knowledge, and political literacy ( Congge et al., 2023; Satria et al., 2023; Efebeh et al., 2024). The relationship is not assumed to be automatic, but the informational and deliberative opportunities created by digital democracy provide a basis for expecting a positive association with political literacy.

Beyond exposure, digital democracy can be understood as a form of opportunity structure. Online spaces reduce some costs of obtaining political information, locating like-minded networks, and responding publicly to issues, but they also require users to decide which sources are credible and which forms of engagement are meaningful. This distinction helps explain why a positive DD-PL association is theoretically plausible without assuming that digital participation is uniformly educational. Digital-democracy scholarship stresses that participatory infrastructures work through institutional design, transparency, and the quality of public communication ( Congge et al., 2023; Weinhardt et al., 2024). Political literacy can therefore be interpreted as one of the individual-level capacities through which citizens make sense of a more open but also more complex political-information environment.

Hypothesis 1 (H1). Digital Democracy is positively associated with Political Literacy.

*2.6.2 Political Literacy (PL) and Political Participation (PP)*

Political literacy can make participation more accessible by helping citizens understand political issues, procedures, and channels for engagement and by supporting internal political efficacy. Empirical evidence among young and first-time voters has reported positive relationships between political literacy or civic education and political participation ( Revalina et al., 2024; Weinberg, 2022). Accordingly, greater political literacy is expected to be associated with higher political participation.

The relationship should nevertheless be interpreted as probabilistic rather than automatic. A broad review of education and political participation shows that educational exposure does not generate identical participatory outcomes across settings, and that the mechanisms connecting learning with participation remain important to specify ( Willeck & Mendelberg, 2022). Related evidence on media literacy indicates that critical engagement with media can support political knowledge and democratic engagement ( Muringa & Adjin-Tettey, 2024). These studies reinforce the expectation of a positive Political Literacy-Political Participation association while also supporting the need to test contextual conditions that may strengthen or weaken its behavioral expression.

Hypothesis 2 (H2). Political Literacy is positively associated with Political Participation.

*2.6.3 Digital Democracy (DD) and Political Participation (PP)*

Digital democracy can lower practical barriers to engagement by expanding access to political information, communication networks, and opportunities for expression and mobilization. Meta-analytic and cross-national evidence generally links digital media use with civic and political participation, although the magnitude and form of the relationship vary across contexts ( Boulianne, 2020; Saud et al., 2023). Research on Indonesian youth also indicates that social networks provide additional channels for political expression and involvement ( Ida et al., 2025). These findings support expecting a positive association between digital democracy and political participation.

The direct DD-PP hypothesis also reflects the fact that participation can arise through routes that do not require extensive political learning. Digital platforms enable low-cost expressive activities, rapid mobilization, interpersonal political discussion, and connections between online and offline networks. Meta-analytic evidence indicates a generally positive association between digital media use and civic or political participation ( Boulianne, 2020), while research on Indonesian youth points to social networks as important spaces for political expression and engagement ( Ida et al., 2025). Accordingly, the model allows Digital Democracy to remain directly associated with Political Participation even after Political Literacy is introduced as a mediator. This specification is important because it avoids treating political literacy as the only route through which digital environments may be related to participatory behavior.

Hypothesis 3 (H3). Digital Democracy is positively associated with Political Participation.

*2.6.4 Political Literacy (PL) as a Mediator*

The mediation argument addresses the mechanism that prior variable-by-variable research does not fully explain. Digital democracy can expose citizens to political information and interaction, but exposure alone does not ensure that these opportunities are translated into participation. Political literacy provides the interpretive knowledge and critical capacity through which digital political experiences can become meaningful for civic action. Contemporary civic-education research connects political knowledge, democratic attitudes, and political self-efficacy with willingness to participate ( Schmitt et al., 2024), while studies of digital democracy emphasize public knowledge and literacy as conditions for meaningful engagement ( Congge et al., 2023; Weinhardt et al., 2024).

This mechanism is also consistent with research showing that digital competencies and political behavior are connected through how individuals interpret and use online information. Guess and Munger (2023) demonstrate that digital literacy is related to online political behavior, while Austin et al. (2021) show that media literacy can shape political engagement in environments marked by disinformation ( Guess & Munger, 2023; Austin et al., 2021). Although these studies examine adjacent forms of literacy rather than the exact mediation tested here, they support the broader proposition that access to digital political information becomes behaviorally meaningful through citizens’ interpretive competencies. Political Literacy is therefore positioned as a theoretically plausible, but empirically testable, intervening mechanism.

Political literacy is therefore proposed as an explanatory mechanism linking digital democracy with political participation: digital environments provide information and interaction opportunities, while political literacy helps citizens interpret those opportunities and connect them with available forms of participation. Because the study uses cross-sectional observational data, this mediation is interpreted as a statistical indirect association rather than proof of a causal process.

Hypothesis 4 (H4). Political Literacy mediates the positive association between Digital Democracy and Political Participation.

*2.6.5 Post-Truth Politics (PT) as a Moderator*

The association between political literacy and participation may depend on the quality and credibility of the surrounding information environment. Under relatively low post-truth conditions, political knowledge and critical evaluation can be more readily connected to political action. Under stronger post-truth conditions, however, contradictory information, distrust, emotionally charged narratives, selective exposure, and persistent misinformation can make it harder for citizens to translate literacy into participation ( Ecker et al., 2022; Vozab, 2023). Post-Truth Politics is therefore theorized as a boundary condition rather than as a substitute for political literacy.

A moderation perspective is particularly useful because the same level of political literacy may have different behavioral implications across information environments. When citizens encounter relatively coherent factual cues and credible sources, political knowledge and interpretive skills can more readily support decisions about whether and how to participate. By contrast, repeated belief-consistent claims, uncertainty about source credibility, and affective polarization may encourage confirmation bias, deepen distrust, and reduce confidence in the informational basis of political action ( Ecker et al., 2022; Vasist et al., 2024). The hypothesis therefore concerns attenuation in the PL-PP association rather than a claim that post-truth politics eliminates the relevance of literacy. This distinction is central to the proposed boundary-condition logic.

Accordingly, the expected moderation is directional: as Post-Truth Politics increases, the positive Political Literacy–Political Participation association is expected to become weaker. This formulation specifies the conditional relationship directly and avoids interpreting the interaction as Political Literacy changing the effect of Post-Truth Politics.

Hypothesis 5 (H5). The positive association between Political Literacy and Political Participation is weaker at higher levels of Post-Truth Politics.

The proposed research model is shown in Figure 1.

**Figure 1.**
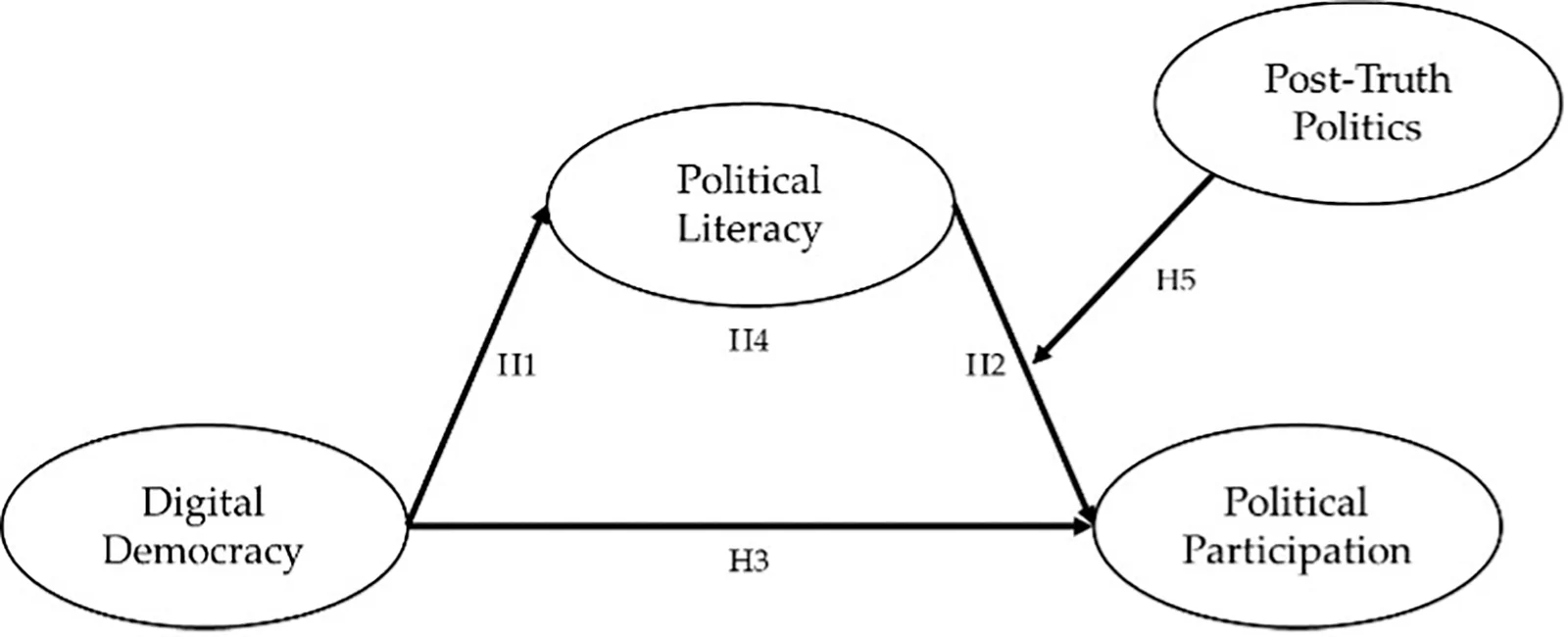
Research model. Source: Constructed by the authors (2025).

## 3. Methodology

### 3.1 Research design, participants, and data collection

This study employed a quantitative cross-sectional survey design to examine the relationships among Digital Democracy (DD), Political Literacy (PL), Post-Truth Politics (PT), and Political Participation (PP) among Generation Z in Indonesia. Participants were recruited through an online survey link using a non-probability purposive sampling approach. Eligible respondents were Generation Z voters aged 18–28 years, residing in Indonesia, and registered in the 2024 Permanent Voter List. The final analytic sample consisted of 500 complete responses from respondents across all 38 provinces. Because recruitment did not use a probability-based provincial sampling frame, coverage of all provinces should be interpreted as geographic coverage rather than population-proportionate sampling.

Sample-size adequacy was assessed using statistical-power considerations. Assuming a medium effect size (f ^2^ = 0.15), α = 0.05, power = 0.95, and four predictors, the minimum required sample was approximately 129 respondents. The final sample of 500 therefore exceeded this requirement.

Ethical approval was obtained from the Graduate School of Universitas Airlangga under approval number 2212/B/UN3.SPS/I/PT.01.05/2025, dated 7 July 2025. Data were collected from August to September 2025 after participants provided informed consent. Because the survey was administered after the 2024 Indonesian general election, items explicitly referring to the 2024 electoral context involved retrospective self-report and may therefore be subject to recall bias.

### 3.2 Measures

The questionnaire consisted of respondent characteristics and 77 substantive items measured using a four-point Likert scale ranging from 1 (strongly disagree) to 4 (strongly agree). Negatively worded items were reverse-scored where necessary before analysis. The complete questionnaire and item wording are provided in the extended data repository described in the Data availability section.

Before data collection, the questionnaire was reviewed by an expert panel in accordance with the applicable instrument-validation procedures of Universitas Airlangga.

Digital Democracy was measured using 25 items adapted primarily from the online political communication and participation measures of Gil de Zúñiga et al. (2010), including online expression, political messaging, political talk, media reflection, and political news use.

Political Literacy was measured using 11 items covering political-information use, political interest, and trust and critical orientation toward political information. The measure was informed by Karim et al. (2015) and adapted in part from Gayatri and Satwika (2022).

Post-Truth Politics was measured using 18 researcher-developed items informed by literature on post-truth politics, misinformation, disinformation, hoaxes, and factual political judgment, including Keyes (2004), Allcott and Gentzkow (2017), Wardle and Derakhshan (2018), and Giusti and Piras (2021).

Political Participation was measured using 23 items adapted primarily from Yolanda and Halim (2020), covering instrumental, informative, and strategic forms of Generation Z online political participation.

### 3.3 Data analysis

Data were analyzed using covariance-based Structural Equation Modeling (CB-SEM) with IBM SPSS AMOS. Prior to model estimation, the data were screened for missing values, coding errors, normality, multivariate outliers, and multicollinearity. Mahalanobis distance was used to identify potential multivariate outliers, variance inflation factors (VIFs) were used to assess multicollinearity, and Harman’s single-factor test was used as an initial diagnostic for potential common-method bias.

The measurement model was assessed through Confirmatory Factor Analysis (CFA) using Maximum Likelihood (ML) estimation. Reliability and validity were evaluated using standardized factor loadings, Cronbach’s alpha, composite reliability (CR), average variance extracted (AVE), and the heterotrait-monotrait ratio (HTMT). Cronbach’s alpha and CR values ≥0.70, AVE ≥0.50, and HTMT <0.85 were used as the primary criteria. Indicators with weak loadings were considered for removal only when supported by both statistical and theoretical considerations. Model fit was evaluated using CMIN/DF, GFI, CFI, TLI, RMSEA, and SRMR, with CMIN/DF ≤ 3.00, GFI/CFI/TLI ≥ 0.90, RMSEA ≤0.08, and SRMR ≤0.08 indicating acceptable fit.

After establishing an acceptable measurement model, the structural model was used to test the hypothesized relationships. Standardized path coefficients, standard errors, test statistics, p-values, and confidence intervals were reported. The mediating role of Political Literacy was assessed using 5,000 bootstrap resamples with 95% confidence intervals. Mediation was considered significant when the confidence interval for the indirect effect did not include zero; significant direct and indirect effects were interpreted as partial mediation, whereas a significant indirect effect with a non-significant direct effect was interpreted as full mediation.

Moderation was tested using standardized composite scores for Political Literacy and Post-Truth Politics rather than a latent-product interaction. The standardized scores were multiplied to create an observed PL × PT interaction term, and Political Participation was modeled as a function of Political Literacy, Post-Truth Politics, and the interaction term. A significant negative interaction coefficient indicated that the positive Political Literacy–Political Participation association was weaker at higher levels of Post-Truth Politics. Significant interactions were further examined using simple-slope analyses at low (−1 SD), mean, and high (+1 SD) levels of Post-Truth Politics and presented using an interaction plot.

## 4. Results

### 4.1 Preliminary analysis and measurement model

A total of 500 complete responses covering all 38 provinces were included in the analysis. No missing values or out-of-range responses were identified. The construct scores showed no severe univariate non-normality, with skewness ranging from 0.006 to 0.116 and kurtosis from −1.123 to −0.976. Multivariate normality was not fully supported by Mardia’s test; therefore, the results were interpreted with appropriate caution and bootstrapping was used for the indirect-effect analysis. No cases exceeded the Mahalanobis-distance criterion at p < .001. Harman’s single-factor test showed that the first factor accounted for 33.62% of the total variance, suggesting that a single common factor did not dominate the data.

The four-factor measurement model was assessed using CFA with Maximum Likelihood estimation. The model showed satisfactory overall fit, χ ^2^(2843) = 3137.43, CMIN/DF = 1.10, CFI = 0.990, TLI = 0.990, RMSEA = 0.014, and SRMR = 0.030. The GFI was 0.865; although below the conventional 0.90 criterion, the remaining fit indices consistently indicated satisfactory model fit.

All 77 indicators were retained, with standardized factor loadings ranging from 0.717 to 0.840. Cronbach’s alpha and composite reliability values exceeded 0.70 for all constructs, while AVE values exceeded 0.50, supporting internal consistency and convergent validity. The detailed results are presented in Table 1.

**Table 1.** Measurement model assessment.

Construct	Items	Loading range	Cronbach’s α	CR	AVE
Digital Democracy	25	0.721–0.840	0.976	0.976	0.618
Political Literacy	11	0.725–0.832	0.946	0.946	0.615
Post-Truth Politics	18	0.741–0.820	0.966	0.966	0.616
Political Participation	23	0.717–0.818	0.971	0.971	0.596

Discriminant validity was examined using the HTMT ratio in Table 2. All values ranged from 0.100 to 0.559, well below the conservative threshold of 0.85, indicating adequate empirical distinction among the four constructs.

**Table 2.** HTMT discriminant validity.

Construct	DD	PL	PT	PP
DD	—	0.512	0.145	0.501
PL	0.512	—	0.164	0.559
PT	0.145	0.164	—	0.100
PP	0.501	0.559	0.100	—

### 4.2 Structural model and hypothesis testing

The structural model showed that Digital Democracy was positively associated with Political Literacy (β = 0.491, SE = 0.039, t = 12.591, p < 0.001), supporting H1. Political Literacy was positively associated with Political Participation while accounting for Digital Democracy (β = 0.390, SE = 0.041, t = 9.425, p < 0.001), supporting H2. Digital Democracy also remained positively associated with Political Participation (β = 0.296, SE = 0.041, t = 7.137, p < 0.001), supporting H3. The standardized path estimates and confidence intervals are presented in Table 3.

**Table 3.** Structural model and hypothesis testing.

Hypothesis	Path	β	SE	t	p	95% CI	Decision
H1	DD → PL	0.491	0.039	12.591	<0.001	[0.415, 0.568]	Supported
H2	PL → PP	0.390	0.041	9.425	<0.001	[0.309, 0.472]	Supported
H3	DD → PP	0.296	0.041	7.137	<0.001	[0.214, 0.377]	Supported

### 4.3 Mediation and moderation results

The mediating role of Political Literacy was examined using 5,000 bootstrap resamples. The indirect association between Digital Democracy and Political Participation through Political Literacy was significant (β = 0.192, 95% CI [0.144, 0.244]), as the confidence interval did not include zero. The direct association between Digital Democracy and Political Participation remained significant (β = 0.296, p < 0.001), indicating partial mediation and supporting H4. The mediation results are presented in Table 4.

**Table 4.** Mediation analysis.

Effect	β	95% CI	Result
DD → PL → PP	0.192	[0.144, 0.244]	Significant
DD → PP (direct)	0.296	[0.214, 0.377]	Significant
Mediation type	—	—	Partial mediation

Post-Truth Politics significantly moderated the association between Political Literacy and Political Participation. The interaction between Political Literacy and Post-Truth Politics was negative and significant (β = −0.173, SE = 0.037, t = −4.665, p < 0.001, 95% CI [−0.246, −0.100]), supporting H5. This negative interaction indicates that the positive association between Political Literacy and Political Participation became weaker as Post-Truth Politics increased. The moderation results are presented in Table 5.

**Table 5.** Moderation analysis.

Predictor	β	SE	t	p	95% CI
Political Literacy	0.535	0.038	14.246	<0.001	[0.461, 0.609]
Post-Truth Politics	0.013	0.038	0.335	0.738	[−0.061, 0.086]
PL × PT	−0.173	0.037	−4.665	<0.001	[−0.246, −0.100]

Simple-slope analysis further showed that the positive association between Political Literacy and Political Participation was strongest when Post-Truth Politics was low and progressively weaker as Post-Truth Politics increased. The simple-slope results are presented in Table 6.

**Table 6.** Simple-slope analysis.

Level of Post-Truth Politics	PL → PP slope	p
Low (−1 SD)	0.709	<0.001
Mean	0.535	<0.001
High (+1 SD)	0.362	<0.001

The VIF values for Political Literacy, Post-Truth Politics, and their interaction ranged from approximately 1.00 to 1.03, indicating no problematic multicollinearity.

Figure 2 shows that the positive association between Political Literacy and Political Participation was weaker at higher levels of Post-Truth Politics. The slope decreased from 0.709 at low Post-Truth Politics to 0.535 at the mean level and 0.362 at high Post-Truth Politics, supporting the negative moderation pattern proposed in H5.

**Figure 2.**
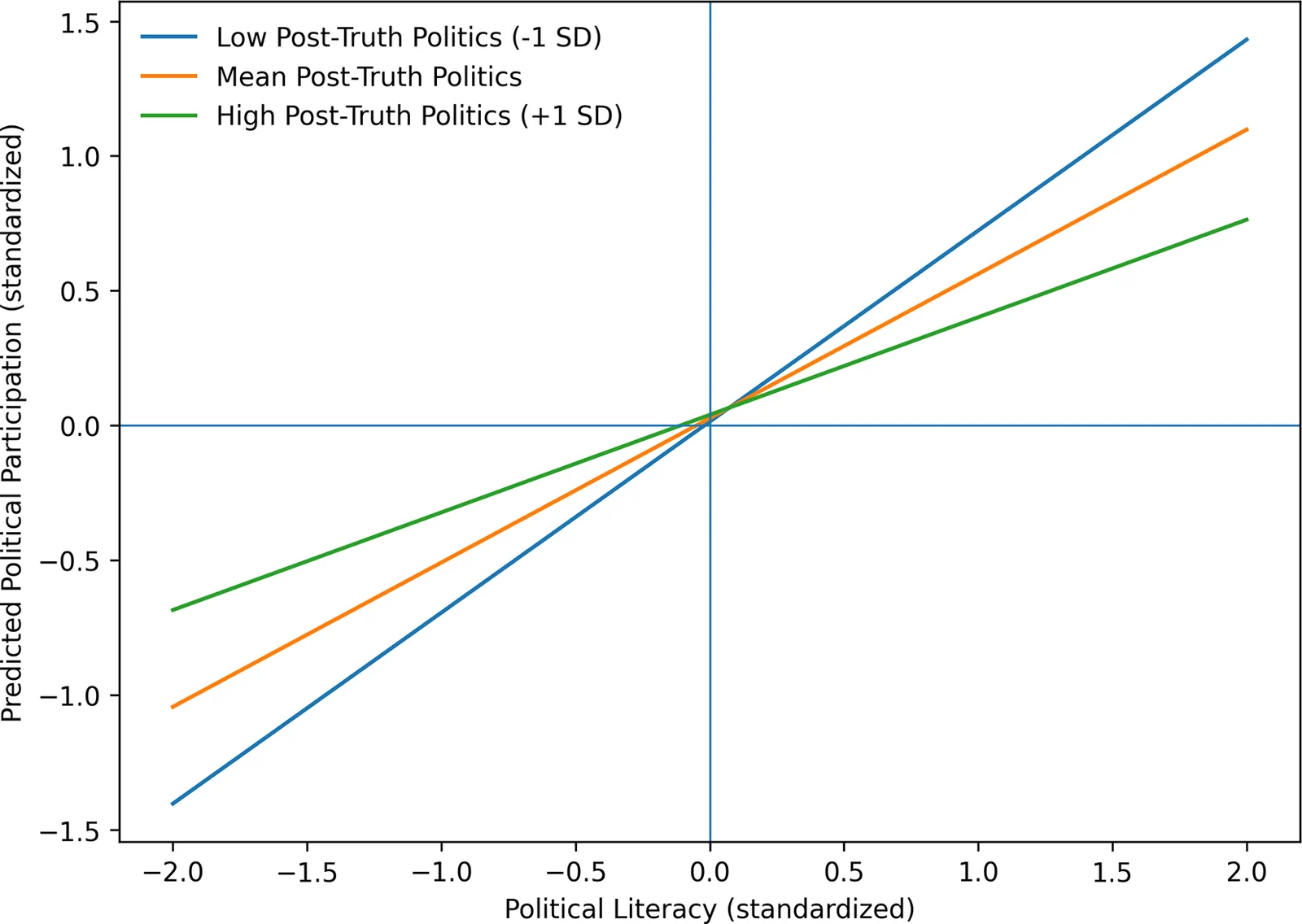
Moderating effect of Post-Truth Politics on the association between Political Literacy and Political Participation.

## 5. Discussion

This study examined how Digital Democracy, Political Literacy, and Post-Truth Politics are associated with Generation Z political participation in the context of the 2024 Indonesian election. The findings show a consistent pattern: Digital Democracy was positively associated with both Political Literacy and Political Participation, Political Literacy was positively associated with Political Participation, Political Literacy partially mediated the Digital Democracy-Political Participation association, and Post-Truth Politics weakened the positive Political Literacy-Political Participation association. Taken together, the results suggest that digital democratic environments are linked to participation both directly and through political literacy, while the surrounding post-truth information environment conditions how strongly political literacy is associated with participation. Because the study used cross-sectional retrospective self-reports, these findings are interpreted as associations rather than causal effects.

### 5.1 Digital democracy and political literacy

The positive association between Digital Democracy and Political Literacy supports H1. This finding is consistent with Satria et al. (2023), who reported that social media can support the political literacy of first-time voters, and Efebeh et al. (2024), who found a positive relationship between social media use and political literacy among eligible voters. It is also compatible with the broader view of Weinhardt et al. (2024) that literacy is an important component of resilient digital democracy. In the present study, greater involvement in digital democratic spaces was associated with greater political literacy among Gen Z respondents.

A plausible interpretation is that digital democratic environments provide repeated opportunities to encounter political information, observe competing viewpoints, discuss public issues, and interact with political communication. Such exposure may create an informal civic-learning environment in which political knowledge and critical orientation can develop. However, the finding should not be read as evidence that digital exposure automatically produces political literacy. Digital environments also contain low-quality and misleading information, so the observed relationship is better understood as evidence that access and participation in digital democratic spaces are linked to political literacy under the conditions captured by this study. This first relationship is important because it establishes the initial stage of the mechanism examined in the mediation model.

The result also fits newer evidence from Indonesia. Borneta et al. (2025) report that Electoral Commission social-media content can support political literacy among Generation Z, suggesting that digitally mediated institutional communication may function as a learning resource. At the same time, the present finding should not be read as evidence that any increase in platform use necessarily produces greater literacy. Digital-democracy research emphasizes that platform design, information quality, and the organization of participation matter for democratic outcomes ( Borneta et al., 2025; Berg & Hofmann, 2021). The contribution of H1 is therefore to show that the positive DD-PL association is observable in a nationwide Generation Z sample while retaining the theoretical distinction between digital access and political understanding.

### 5.2 Political literacy and political participation

Political Literacy was positively associated with Political Participation while accounting for Digital Democracy, supporting H2. This result is consistent with Revalina et al. (2024), who found a positive relationship between political literacy and participation among first-time voters, and with evidence from civic-education research showing that participatory and discussion-based learning can be linked to greater political engagement ( Weinberg, 2022). The result therefore supports the view that political literacy is a relatively proximal correlate of participation among young voters.

Political literacy may matter because understanding political issues, procedures, and available channels of participation can reduce the cognitive barriers associated with political engagement and make participation more intelligible and personally relevant. This interpretation is also compatible with Noprianto, Wantu, and Djaafar (2025), who linked political education with voter participation. Nevertheless, the present analysis does not directly demonstrate that politically literate respondents were better at avoiding misinformation or that their participation was of higher democratic quality. The result is more narrowly interpreted as showing that higher Political Literacy scores were associated with higher reported Political Participation.

This interpretation is compatible with recent work linking civic and media literacy to democratic engagement. Muringa and Adjin-Tettey (2024) associate media literacy with political knowledge, critical analysis, and engagement among university students, while Willeck and Mendelberg (2022) caution that the education-participation relationship varies according to mechanisms and context ( Muringa & Adjin-Tettey, 2024; Willeck & Mendelberg, 2022). For the present study, this helps explain why Political Literacy remained significantly associated with participation even after Digital Democracy was included in the structural model. The result is consistent with literacy operating as a more proximal interpretive resource rather than merely reflecting exposure to political information.

### 5.3 Digital democracy and political participation

Digital Democracy also remained positively associated with Political Participation after Political Literacy was included in the model, supporting H3. This finding is consistent with the meta-analytic evidence reported by Boulianne (2020) and with studies showing that digital and social-media environments are associated with youth political communication and engagement ( Saud et al., 2023; Ida et al., 2025). Digital platforms can provide relatively accessible channels for obtaining political information, expressing views, joining discussions, and responding to public issues, which may help explain why stronger Digital Democracy scores were associated with greater participation.

The coexistence of a significant direct association and a significant indirect association is substantively informative. It suggests that digital democratic environments are related to participation through both literacy-linked and other pathways. For example, social networks may provide invitations to act, visibility of peers’ political behavior, or immediate opportunities for expression that do not depend entirely on increased political knowledge. This interpretation is consistent with the civic-voluntarism emphasis on resources, engagement, and recruitment networks ( Brady et al., 1995) and with research showing that digital media can broaden repertoires of participation ( Norris, 2002). The H3 result therefore complements, rather than competes with, the mediation finding.

Importantly, the direct Digital Democracy-Political Participation association remained significant even after Political Literacy was considered. This indicates that political literacy does not account for the entire relationship. Other processes proposed in the digital-participation literature, such as mobilization, recruitment through online networks, political efficacy, or lower participation costs, may also contribute to the direct association, although these mechanisms were not measured in this study. The result therefore supports a multi-path interpretation in which digital democracy is associated with participation through both literacy-related and other unobserved pathways.

### 5.4 The mediating role of political literacy

The mediation analysis provides the central explanatory finding of the study. The indirect association between Digital Democracy and Political Participation through Political Literacy was significant (β = 0.192, 95% CI [0.144, 0.244]), while the direct association remained significant (β = 0.296). Political Literacy therefore showed partial mediation, supporting H4. In substantive terms, part of the association between Digital Democracy and Political Participation is linked to the extent to which digital democratic engagement is also associated with political literacy.

This pattern clarifies the mechanism more precisely than a simple direct-effect model. Digital democratic spaces may provide information and interaction opportunities, but political literacy represents a cognitive resource through which those opportunities can be understood and related to political action. Related research has similarly emphasized the importance of literacy-related competencies in connecting media environments with civic and political behavior. For example, Erdem et al. (2023) identified a mediating role for digital literacy in a digital-citizenship model, while Lee, Cho, and Kim (2023) showed that media-literacy-related processes are associated with pathways to youth political participation. Although these constructs are not identical to political literacy, they support the broader interpretation that literacy can operate as an intervening mechanism between digital exposure and civic behavior.

The partial, rather than full, mediation is theoretically important. Political Literacy appears to be one mechanism linking Digital Democracy with Political Participation, but not the only mechanism. This avoids treating political literacy as a complete explanation and is consistent with the remaining direct association observed in the structural model.

The partial-mediation pattern also helps refine the contribution of the study. If Political Literacy had fully accounted for the DD-PP association, the model would imply that the relationship between digital democracy and participation operates predominantly through a literacy mechanism. Instead, the retained direct association indicates a more plural process in which literacy explains a meaningful part, but not all, of the relationship. This is consistent with research showing that youth political participation is shaped by multiple informational, social, motivational, and network-based processes ( Lee et al., 2023; Weiss, 2020). Accordingly, Political Literacy is best described as a partial explanatory mechanism rather than as an exclusive transmission channel.

### 5.5 The moderating role of post-truth politics

Post-Truth Politics significantly moderated the Political Literacy-Political Participation association, supporting H5. The interaction coefficient was negative (β = −0.173, p < 0.001), and the simple-slope analysis showed a clear attenuation pattern: the Political Literacy-Political Participation slope was 0.709 when Post-Truth Politics was low, 0.535 at the mean level, and 0.362 when Post-Truth Politics was high. Thus, Political Literacy remained positively associated with Political Participation at all three levels, but the association became progressively weaker as Post-Truth Politics increased.

This finding should be distinguished from a direct effect of Post-Truth Politics. Its main effect on Political Participation was not statistically significant (β = 0.013, p = 0.738). The result therefore does not show that higher Post-Truth Politics simply corresponds to lower participation for all respondents. Rather, it indicates a boundary condition: the strength of the positive Political Literacy-Political Participation relationship depends on the level of Post-Truth Politics. This interpretation directly reflects the conditional relationship specified in H5.

A plausible explanation is that a post-truth information environment introduces conflicting claims, emotionally charged narratives, selective exposure, distrust, and uncertainty about source credibility. These conditions may encourage confirmation bias, in which belief-consistent claims are accepted more readily than competing evidence, and may deepen political cynicism or distrust toward political actors and institutional media. In campaign environments, repeated or strategically framed messages can also operate in propaganda-like ways by shaping attention and perceptions of candidates and issues rather than merely conveying verifiable facts ( Corner, 2017; Giusti & Piras, 2021; Ecker et al., 2022). Such processes may make political knowledge more difficult to translate into participatory behavior, while disinformation can additionally intensify polarization ( Vasist et al., 2024). However, the present survey did not separately measure confirmation bias, cynicism, distrust, populist communication, or propaganda; these should therefore be treated as plausible explanatory mechanisms rather than empirically established effects of the present study.

The moderation pattern is also consistent with work showing that literacy operates within, rather than outside, the surrounding information ecology. Austin et al. (2021) link media literacy with political engagement in a misinformation context, and Lilja et al. (2024) frame civic literacy as part of democratic resilience to disinformation ( Austin et al., 2021; Lilja et al., 2024). The present study extends this logic by showing a conditional association: the PL-PP slope remained positive at all examined levels of Post-Truth Politics, but its magnitude declined as Post-Truth Politics increased. This nuance is important because it suggests attenuation rather than reversal; political literacy remains relevant, but its association with participation is less pronounced in a more post-truth-oriented environment.

The moderation result also qualifies a potentially overgeneralized assumption that political literacy will translate into participation equally across information environments. Political literacy remained relevant even under high Post-Truth Politics, but its association with participation was substantially weaker. In this sense, Post-Truth Politics operates as a contextual boundary condition rather than as evidence that political literacy becomes ineffective.

This qualification has practical as well as theoretical significance, although the cross-sectional design requires caution. If literacy is associated with participation less strongly under high post-truth conditions, then interventions focused only on individual knowledge may be insufficient when the wider information environment remains highly distorted or distrustful. Digital-democracy frameworks increasingly emphasize information integrity, transparency, and accountable platform governance alongside citizen capabilities ( OECD, 2024; Weinhardt et al., 2024). The study’s moderation result is compatible with that broader view: democratic participation is related not only to what citizens know, but also to the informational conditions under which that knowledge is translated into action.

Overall, the findings contribute more than a combination of four variables. The model identifies Political Literacy as a partial explanatory mechanism linking Digital Democracy with Political Participation and Post-Truth Politics as a boundary condition that changes the strength of the literacy-participation association. This mechanism-and-boundary-condition interpretation helps explain both how digital democracy is related to Gen Z political participation and the information context in which the role of political literacy becomes weaker. At the same time, the cross-sectional and retrospective design means that the temporal and causal ordering of these relationships cannot be established. Longitudinal or experimental research is needed to test whether changes in digital democratic engagement precede changes in political literacy and participation and to identify the processes underlying the observed moderation.

## 6. Conclusions

This study examined the associations among Digital Democracy, Political Literacy, Post-Truth Politics, and Political Participation among Generation Z voters in Indonesia. Digital Democracy was positively associated with Political Literacy and Political Participation, and Political Literacy was positively associated with Political Participation. Political Literacy partially mediated the Digital Democracy–Political Participation association, indicating that the relationship between digital-democratic engagement and participation is explained in part through political literacy. Post-Truth Politics negatively moderated the Political Literacy–Political Participation association: the positive association remained significant but became weaker as Post-Truth Politics increased.

These findings clarify the model’s theoretical contribution by identifying Political Literacy as a partial explanatory mechanism and Post-Truth Politics as a contextual boundary condition. Rather than treating digital democracy as directly translating into participation, the results suggest that part of its statistical association with participation operates through political literacy, while the strength of the literacy-participation relationship varies across the post-truth information environment. Because the study is cross-sectional and observational, these findings should be interpreted as associations rather than causal effects.

Practically, the findings suggest that expanding digital channels for civic and political engagement should be accompanied by efforts to strengthen political literacy and information integrity. For election-management bodies, educators, civil-society organizations, news organizations, and digital-platform stakeholders, this includes credible and accessible election information, critical evaluation skills, transparent political-advertising and sponsorship practices, accessible fact-checking and correction mechanisms, safeguards against coordinated misinformation and emotionally manipulative content, and greater transparency around campaign targeting and content-recommendation systems. These measures should complement rather than replace citizen-level literacy, because democratic resilience depends on both individual evaluative capacity and the quality of the information environment in which political judgments are formed.

## 7. Limitations

This study has several limitations. First, the cross-sectional design does not establish temporal or causal ordering among the constructs. Second, data were collected in August–September 2025 about aspects of participation in the 2024 election, so retrospective self-report may be affected by recall bias. Third, all focal variables were measured using the same self-report questionnaire, creating potential common-method variance and response bias; although Harman’s single-factor test did not indicate a dominant common factor, this diagnostic does not eliminate the possibility of method effects. Fourth, respondents were recruited through non-probability purposive online sampling. Coverage of all 38 provinces therefore should not be interpreted as population representativeness, and generalization beyond the observed sample should be made cautiously.

Future research should use longitudinal or panel designs that measure political literacy and participation closer to electoral events, probability-based or more systematically stratified sampling where feasible, and behavioral or multi-source measures to reduce reliance on self-report data. Experimental or quasi-experimental designs could test the direction of the proposed mechanisms more directly, while replication across generations, electoral contexts, and countries could assess the generalizability of the model. Further validation of the Post-Truth Politics measure and direct examination of perceived credibility, source trust and distrust, confirmation bias, selective exposure, information overload, and political cynicism would also strengthen understanding of the observed moderation.

## Ethical approval

This study involved human participants and was conducted in accordance with the Declaration of Helsinki and the ethical guidelines for research involving human respondents at Universitas Airlangga. Ethical approval was obtained from the Graduate School of Universitas Airlangga, approved by the Vice Director I, Prof. Dr. Rudi Purwono, S.E., M.SE, under approval number 2212/B/UN3.SPS/I/PT.01.05/2025, dated 7 July 2025. All research procedures were designed to protect participants’ rights, confidentiality, anonymity, and voluntary participation.

## Informed consent

Informed consent was obtained from participants before data collection. Participants were informed about the purpose of the study, the voluntary nature of their participation, the confidentiality of their responses, and their right to withdraw from the study. For the expert panel involved in the research process, written informed consent was obtained. For the questionnaire survey, participants provided consent before completing the questionnaire. No personally identifiable images were collected in this study, and all responses were treated confidentially and used only for research purposes.

## Data Availability

The underlying data supporting the findings of this study are available on Figshare under the title “Underlying_Data_Anonymized.xlsx” with the following DOI: https://doi.org/10.6084/m9.figshare.32833550.v1 ( Latuconsina, 2026b). The dataset includes anonymized survey responses, variable descriptions, and data used for the statistical analyses reported in this article. All personal identifiers were removed before public sharing. Data are available under the terms of the Creative Commons Attribution 4.0 International license. The extended data for this study are available on Figshare under the title “Questionnaire Instrument for the Study on Digital Democracy, Political Literacy, Post-Truth Politics, and Generation Z Political Participation in the 2024 Indonesian Election” with the following DOI: https://doi.org/10.6084/m9.figshare.32833664.v1 ( Latuconsina, 2026a). The extended data include the questionnaire instrument used to measure Digital Democracy, Political Literacy, Post-Truth Politics, and Political Participation among Generation Z voters in the 2024 Indonesian election. Data are available under the terms of the Creative Commons Attribution 4.0 International license.
