# Supplementary material for: Digital Democracy and Gen Z Political Participation in Indonesia's 2024 Election: The Mediating Role of Political Literacy and the Moderating Role of Post-Truth Politics
Source: F1000Res. 2026 Oct 17;15:1148. Originally published 2026 Jul 13. [Version 2] doi: 10.12688/f1000research.185707.2 (PMC13743843; doi:10.12688/f1000research.185707.2)
Supplement: Peer review history

# Reviewer report: Reviewer response for version 2

**DOI:** 10.5256/f1000research.211063.r531720

**Published:** 2026-11-06

**Authors:** Ajeng Widya Prakasita

**Affiliations:** 1 State Islamic University (UIN) Sunan Ampel Surabaya, Surabaya, Indonesia

Summary of the article: 

 The study examines how Digital Democracy (DD), Political Literacy (PL), Post-Truth Politics (PT) and Political Participation (PP) relate among Indonesian Generation Z voters (aged 18–28) in the context of the 2024 election. It uses an online, purposive, non-probability survey (N = 500, all 38 provinces, collected August–September 2025, 77 four-point Likert items) and CB-SEM in AMOS.

 The authors report that:

- DD is positively associated with PL (β = 0.491) and PP (β = 0.296).
- PL is positively associated with PP (β = 0.390).
- PL partially mediates DD→PP (indirect β = 0.192, 95% CI [0.144, 0.244]).
- PT negatively moderates PL→PP (β = −0.173; simple slopes 0.709, 0.535 and 0.362 at −1 SD, mean and +1 SD of PT).

The authors state that the design is cross-sectional and the findings are associations. The revision is clearer and more cautious than a typical first submission, and the reporting is more transparent. The core problems below concern construct validity, the moderation procedure, confounding, and sampling.

 Here are some of the detailed comments for each part, which partially answer: 

 1. Is the work clearly and accurately presented, and does it cite the current literature? **Partly**

 The literature is extensive and recent, and the writing is clear. However:

- The post-truth concept is anchored in a dictionary definition (Oxford Languages, 2016) rather than scholarly sources (e.g., Lewandowsky et al., 2017; McIntyre, 2018).
- The filter-bubble claims are presented as settled and cited to Dahlgren (2013). Empirical work on filter bubbles and echo chambers is mixed (e.g., Dubois & Blank, 2018; Bruns, 2019).
- Contrary evidence is missing. Motivated-reasoning research (Taber & Lodge, 2006; Kahan et al., 2017) suggests that higher political sophistication can intensify biased processing rather than buffer against it. This directly challenges the assumed role of PL.
- Indonesia-specific work on algorithmic enclaves, buzzers, and the 2024 digital campaigns (e.g., Lim, 2017; Tapsell, 2017) is underused.
- The internet-penetration figure (79.5%) and the Gen Z share are attributed to the Ministry of Communication and Digital Affairs. To my knowledge they come from APJII’s 2024 survey, so the attribution should be verified.
- Section 2.3 devotes much space to mechanisms (confirmation bias, trust, propaganda) that the authors admit were not measured.

2. Is the study design appropriate and is the work technically sound? **Partly**

 The cross-sectional design cannot support the causal chain DD → PL → PP. Main concerns:

- **Construct overlap:** DD is defined as an environment but measured as individual online political behaviour, close to the online-PP outcome. PL has no knowledge items and is built from political interest and information use, classic antecedents of PP. The researcher-developed PT scale does not specify what it captures.
- **Confounding and reverse causation:** no covariates are included, and participation may itself raise literacy.
- **Timing:** data were collected 18–19 months after the election, with unclear reference periods for each construct.
- **Sampling:** purposive online recruitment with no demographic or provincial breakdown. The Acknowledgments thank Partai Demokrat, a potential competing interest and source of partisan bias.
- **Sample size:** the power analysis is for regression, not SEM, and N = 500 for 77 indicators is modest.

3. Are sufficient details of methods and analysis provided to allow replication? **Partly**

 Replication is limited by missing detail:

- Recruitment channels, response numbers, exclusions, and data-quality checks.
- Translation and expert-panel procedures and the wording of the PT scale.
- The exact moderation specification and the AMOS files or syntax.
- Whether the standardized SEs and t-values are bootstrapped.

4. Is the statistical analysis and its interpretation appropriate? **Partly**

 The arithmetic is internally consistent; the concerns are about model specification:

- **Moderation:** it uses observed composites outside the SEM, ignoring measurement error and omitting DD (hence PL = 0.535 versus 0.390). Use a latent interaction and report moderated mediation.
- **Estimator:** four-category, non-normal items are estimated with ML; use WLSMV or robust ML.
- **Unusually clean results:** CFI = 0.990, RMSEA = 0.014, all loadings 0.717–0.840, kurtosis ≈ −1. Request item-level descriptive, correlations, and AMOS output, and inspect the dataset.
- **Common-method variance:** Harman’s test is insufficient; add a latent method factor or marker variable.
- **Missing reporting:** R², interaction effect size, means, and SDs.

5. Are all the source data underlying the results available? **Partly**

 Anonymized data and the questionnaire are on Figshare (CC-BY), but no analysis code or AMOS files are provided. Confirm the file has item-level data, a data dictionary, and the province variable. With code added, this becomes “Yes.”

 6. Are the conclusions drawn adequately supported by the results? **Partly**

 Associational wording is much improved, but “explained in part,” “mechanism,” and “weakened by post-truth” still imply causation, and the practical recommendations go beyond the data. The boundary-condition claim rests on a researcher-developed PT measure and a composite-based interaction, so it should be stated tentatively.

Is the work clearly and accurately presented and does it cite the current literature?

Partly

If applicable, is the statistical analysis and its interpretation appropriate?

Partly

Are all the source data underlying the results available to ensure full reproducibility?

Partly

Is the study design appropriate and is the work technically sound?

Partly

Are the conclusions drawn adequately supported by the results?

Partly

Are sufficient details of methods and analysis provided to allow replication by others?

Partly

Reviewer Expertise:

My areas of research are urban politics, big data and digital politics, and local politics

I confirm that I have read this submission and believe that I have an appropriate level of expertise to confirm that it is of an acceptable scientific standard, however I have significant reservations, as outlined above.

# Reviewer report: Reviewer response for version 2

**DOI:** 10.5256/f1000research.211063.r531050

**Published:** 2026-10-22

**Authors:** Pradeep Nair

**Affiliations:** 1 Central University of Himachal Pradesh, Dharamshala, Himachal Pradesh, India; 2 Sabin Center for Climate Change Law, Columbia University (Ringgold ID: 5798), New York, New York, USA

Despite the increasing role of digital media in active political governance and the policy domain, its socio-political consequences remain less explored. There is no doubt that in recent times, digital media platforms have become a crucial force in the contemporary political world, with more insights on how Gen Z accesses and engages with political information in their day-to-day life. A large number of young people worldwide have their dependency on digital media for access to news and information. This overwhelming response to digital media as the most preferred platform for communication has spurred considerable scholarly interest in defining the role of digital media in modern democracy. This study provides new insights on how digital media proves itself as an effective tool for accomplishing political goals by raising public awareness, changing young people’s minds about issues, and getting elected officials to pay attention to issues influencing policy decisions in the context of Indonesia’s General Elections held in 2024.

Is the work clearly and accurately presented and does it cite the current literature?

Yes

If applicable, is the statistical analysis and its interpretation appropriate?

Not applicable

Are all the source data underlying the results available to ensure full reproducibility?

Partly

Is the study design appropriate and is the work technically sound?

Partly

Are the conclusions drawn adequately supported by the results?

Partly

Are sufficient details of methods and analysis provided to allow replication by others?

Partly

Reviewer Expertise:

digital democracy, political participation, governance and policy research

I confirm that I have read this submission and believe that I have an appropriate level of expertise to confirm that it is of an acceptable scientific standard.

# Reviewer report: Reviewer response for version 1

**DOI:** 10.5256/f1000research.204974.r519796

**Published:** 2026-09-24

**Authors:** Pradeep Nair

**Affiliations:** 1 Central University of Himachal Pradesh, Dharamshala, Himachal Pradesh, India; 2 Sabin Center for Climate Change Law, Columbia University (Ringgold ID: 5798), New York, New York, USA

The study leverages digital information systems research in the context of political literacy to advance the study of digital democracy. The interdisciplinary approach of the study examines how digital platforms and technologies influence the political participation of Gen Z in the 2024 general elections in Indonesia, with a focus on their role in polarization, disinformation, post-truth, and democratic processes. By integrating data-driven analysis, the study aims to deliver actionable insights for fostering more resilient digital democracies in the context of general elections and political governance in South Asia.

 The initial focus of the study is on the theoretical underpinnings of the post-truth phenomenon, its emergence and development, as well as its practical realization and impact on Gen Z political participation in the 2024 general elections in Indonesia. The study looks into how the belief systems shape an individual’s perception of politics and governance in the era of digital democracy and how the data-driven political campaigns manipulate public understanding of general elections by and large. The study also explores how the Gen Z citizens seek confirmation bias as opposed to interrogating the claims and counterclaims of political campaigns in the context of 2024 general elections held in Indonesia.

 The ideas of post-truth politics are largely situated in paranoia whereby “departures from truth are lies, delusions, and phantasy." In the context of the political events of Indonesia, the study urges that the populist political outpourings are marked by misinformation and manipulation of truth that result from the cultural shift in the conception of ‘self’ that is both corroding the core pillars of the civilizing process and altering the nature of youth engagement with democratic politics, which has led to the destabilization of the contemporary notions of democracy in most of the general elections in South Asian countries.

 In this study there is a need to have a better weaving of the analysis and arguments of the existing gaps in understanding these concepts into a more practical frame. There is a need to explore how political communication processes have challenged traditional notions of truth in the age of digital democracy. In charting changes in the approach to political literacy, there is a dire need to redefine post-truth to suit the idea of digital democracy. Right now, there is limited construction of post-truth in terms of general elections and governance, and most of the post-truth narratives in contemporary politics are engaged with cross-checking facts when reporting political communication.

 Whereas, the focus shall be on how information about the elections, manifestos, candidates, and leadership is presented along with the trust arguments, which draw on selective data, blindly relying on the emotions of Gen Z irrespective of the facts. Arguably, this can go beyond the demonization of opponents and leads to multiple distorted perceptions of reality that can lead to increased marginalization through stereotyping and routinizing.

 The study design lacks essential details. The whole study is presented loosely, and connecting points were missing. Further, what is missing is the theoretical contribution that the research forwards. How trust is built in political participation and its further expansion through media interventions and social mechanisms is not sufficiently addressed in the study and needs substantial evidential support. The phenomenon of the post-truth, fake news social media environment needs more detailed mention in the study. The notion of post-truth entails more than fabrication but also actively seeks confirmation bias. Describing “post-truth as a symptom rather than a cause,” there is a need to argue that although post-truth is not a new phenomenon and lies by politicians are nothing new, people are bombarded by fake information online, leading the distinction between facts and fiction to become increasingly blurred as blogs, social media, citizen journalism, etc., are awarded similar credibility to other information sources.

 Interestingly, the study argues how fake news and post-truth are “two interwoven phenomena that serve specific financial or ideological interests.” A wider representation of the reinvention of propaganda, showing propaganda and post-truth to be means of shaping public opinion, needs to be discussed further. In the context of Gen Z’s political participation, an obsession with the spectacle of politics, an underlying cynicism towards politicians, an inadequate model for dealing with issues where the balance of evidence is not equal, and a deepening distrust of the mainstream media encouraged by populist outsiders, further discussion is needed.

 The study contextualized post-truth as contributors to atmospheres of irrational fears and hopes leading to citizens feeling disempowered and disillusioned and so fertile for manipulation by the next populist outsider’s post-truth campaign. Media literacy in political participation was highlighted as one panacea, as well as tighter regulation over media and political uses of language and rhetoric. Post-truth, participants agreed, was not known, and the term, if not inherently useful, however, its emergence has brought renewed focus on dishonesty in democracy. There is a need for Gen Z citizens to be better equipped to judge veracity within the complex 21st-century digital communication environment.

Is the work clearly and accurately presented and does it cite the current literature?

Yes

If applicable, is the statistical analysis and its interpretation appropriate?

Not applicable

Are all the source data underlying the results available to ensure full reproducibility?

Partly

Is the study design appropriate and is the work technically sound?

Partly

Are the conclusions drawn adequately supported by the results?

Partly

Are sufficient details of methods and analysis provided to allow replication by others?

Partly

Reviewer Expertise:

digital democracy, political participation, governance and policy research

I confirm that I have read this submission and believe that I have an appropriate level of expertise to confirm that it is of an acceptable scientific standard, however I have significant reservations, as outlined above.

# Response

**Published:** 2026-10-14

**Authors:** Agust Jovan Latuconsina

**Affiliations:** Universitas Airlangga Departemen Politik, Surabaya, East Java, Indonesia

Thank you very much for your thoughtful and constructive comments. In response, we have further strengthened the manuscript by providing a more integrated theoretical and practical explanation of post-truth politics within digital democracy and Generation Z political participation. The revised manuscript now clarifies that post-truth is not limited to the circulation of false information, but represents a broader information environment in which emotion, identity, prior beliefs, selective exposure, and perceived source credibility may become more influential than factual verification. We have therefore expanded the discussion to incorporate confirmation bias, trust and distrust, selective exposure, emotionally framed political communication, and the ways in which digital political campaigns may shape perceptions of candidates, manifestos, leadership, and electoral choices. We have also distinguished post-truth politics from related concepts such as misinformation, disinformation, fake news, and propaganda, explaining that these may operate as communicative mechanisms that contribute to a wider post-truth environment. In addition, the Discussion has been strengthened by considering political cynicism, declining trust in institutional or mainstream media, populist-style communication, and affective polarization as theoretically plausible processes that may help explain why the association between Political Literacy and Political Participation becomes weaker under stronger post-truth conditions. Because these mechanisms were not separately measured in the present study, they are explicitly presented as interpretive explanations rather than direct empirical findings.

 We have also made the study’s theoretical contribution more explicit by positioning Political Literacy as a partial explanatory mechanism linking Digital Democracy with Political Participation and Post-Truth Politics as a contextual boundary condition that weakens the Political Literacy–Political Participation association. This framing moves the contribution beyond merely combining several variables and instead emphasizes the mechanism through which digital democratic engagement is associated with participation and the information conditions under which that relationship becomes less pronounced. The practical implications have likewise been expanded to emphasize political and media literacy, credible election information, information integrity, accessible fact-checking, transparency in political advertising and campaign targeting, and greater platform accountability. The methodological description had already been substantially strengthened in the revised version, including clarification of the cross-sectional and retrospective design, purposive sampling, instrument development, SEM procedures, mediation and moderation analysis, and the limitation that the findings represent statistical associations rather than causal effects. Finally, while we appreciate the reviewer’s broader regional perspective, we have retained the empirical framing around Indonesia because the study does not provide comparative evidence that would justify generalization to South Asia. All corresponding corrections have been marked directly on the PDF proof using commenting and markup annotations, in accordance with the editor’s instructions.

# Reviewer report: Reviewer response for version 1

**DOI:** 10.5256/f1000research.204974.r519790

**Published:** 2026-09-15

**Authors:** Aisyah Aryandani

**Affiliations:** 1 Ilmu Komputer, Universitas Islam Al Azhar, Mataram, West Nusa Tenggara, Indonesia

This article examines the relationships among digital democracy, political literacy, post-truth politics, and political participation among Generation Z voters in Indonesia in the context of the 2024 general election. The authors propose that digital democracy positively influences political literacy and political participation, that political literacy positively influences political participation and mediates the relationship between digital democracy and political participation, and that post-truth politics negatively moderates the relationship between political literacy and political participation.

 The topic is timely and relevant, particularly given the increasing importance of social media, misinformation, digital political communication, and young voters in contemporary electoral politics. The manuscript also incorporates a substantial body of literature, including several recent references published between 2023 and 2026. The proposed integration of political literacy as a mediator and post-truth politics as a moderator is potentially useful.

 The empirical analysis is based on 534 Generation Z respondents, and the authors use covariance-based Structural Equation Modeling with AMOS. The reported model-fit statistics are generally acceptable (CMIN/DF = 2.14, GFI = 0.92, CFI = 0.95, TLI = 0.94, and RMSEA = 0.056). The constructs also report Cronbach's alpha, composite reliability, and AVE values exceeding conventional minimum thresholds.

 However, several methodological, analytical, and reporting issues should be addressed before the manuscript can be considered scientifically robust.

**1. Presentation and engagement with the literature — Partly**

 The manuscript contains an extensive literature review and importantly includes contemporary studies on digital democracy, misinformation, political literacy, and youth political participation. Therefore, the literature base is generally relevant and reasonably current.

 Nevertheless, the presentation requires greater precision and consistency. There are several places where broad normative statements are presented as empirical facts without sufficient evidence, particularly in the Introduction and Discussion. The manuscript would benefit from distinguishing more carefully between normative claims about democracy and empirically established relationships.

 The research gap also needs to be formulated more rigorously. The manuscript currently emphasizes that few studies simultaneously examine the four variables. Merely combining previously studied variables is not, by itself, a sufficiently strong theoretical contribution. The authors should explain what theoretical mechanism is unresolved in prior research and why political literacy specifically functions as a mediator and post-truth politics specifically functions as a boundary condition.

 The theoretical framework should therefore move beyond a “variable combination” rationale and explain the underlying causal or behavioral mechanisms.

**Required revision:** strengthen the theoretical gap and explicitly identify what previous studies have failed to explain, rather than relying primarily on the novelty of combining the variables.

**2. Study design and technical soundness — Partly**

 A quantitative survey and SEM are reasonable approaches for examining relationships between latent constructs. However, the present design has several important limitations.

 First, the manuscript uses causal language such as “influence,” “strengthens,” and “affects,” despite relying on a non-experimental, cross-sectional observational survey. Such a design can provide evidence of statistical associations but cannot establish causal effects. The wording throughout the manuscript, including the title, hypotheses, discussion, and conclusions, should therefore be reconsidered or the causal interpretation explicitly qualified.

 Second, there is an important timing issue. The study concerns political participation in the **2024 election**, yet participant recruitment occurred in **August–September 2025**.

 Thus, the study appears to rely at least partly on retrospective self-reports collected approximately one year after the electoral event. This creates possible recall bias and raises an important question regarding what exactly was measured: actual participation during the 2024 election, current political participation, or respondents' retrospective perceptions of their 2024 participation. This distinction needs to be clearly specified.

 Third, there is an internal inconsistency regarding geographic coverage. The Abstract states that respondents were registered voters across **38 provinces**, whereas the Methodology states that the sample was selected across **34 provinces**.

 This discrepancy must be corrected and the exact sampling coverage reported.

 Fourth, although the authors describe the procedure as “proportionate stratified sampling,” there is insufficient information about how individual respondents were actually selected within each province. A proportional geographic allocation does not automatically constitute probability sampling. The authors need to report the sampling frame, recruitment procedure, selection mechanism within strata, response rate, inclusion/exclusion criteria, and how the provincial allocation was determined.

 The manuscript should also explain the basis for the sample size of 534 respondents, preferably through power analysis, SEM sample-size considerations, or another a priori justification.

**These sampling issues should be considered major revisions because they directly affect the generalizability of the findings.**

**3. Methods and reproducibility — Partly**

 The manuscript provides a general description of the research design, constructs, four-point Likert scale, SEM-AMOS analysis, model-fit indices, and bootstrapping.

 However, insufficient methodological detail is currently presented for a fully reproducible analysis.

 The authors should provide much clearer information regarding:

- the theoretical source or previous validated scale from which each item was derived;
- whether questionnaire items were adopted, adapted, translated, or newly developed;
- translation/back-translation procedures if applicable;
- pretesting or pilot testing;
- the expert-review procedure;
- item deletion or retention criteria;
- standardized CFA factor loadings;
- residuals and/or modification procedures, if used;
- assessment of multivariate normality and outliers;
- treatment of missing data;
- estimator used in AMOS;
- bootstrap sample size and confidence intervals;
- discriminant validity;
- multicollinearity;
- and possible common-method bias.

This is particularly important because the measurement model is relatively large: the manuscript reports **24 indicators for Digital Democracy, 11 for Political Literacy, 18 for Post-Truth Politics, and 23 for Political Participation**, totaling 76 indicators.

 With such a large measurement model, reporting only CA, CR, and AVE is insufficient to evaluate measurement quality.

 The public availability of the questionnaire is helpful, but the manuscript itself should still explain how the instrument was developed and validated.

**4. Statistical analysis and interpretation — Partly**

 The general choice of SEM is defensible, and the reported global model-fit indices appear acceptable.

 The structural estimates reported are:

 H1: DD → PL, β = 0.61, p < .001

 H2: PL → PP, β = 0.48, p < .001

 H3: DD → PP, β = 0.37, p < .001

 H4: DD → PL → PP, indirect effect = 0.29, p = .002

 H5: PL × PT → PP, β = −0.21, p = .006.

 Several points nevertheless require revision.

**Most importantly, the moderation analysis is insufficiently documented.** The authors should explain how the interaction term between political literacy and post-truth politics was constructed in AMOS, whether variables were mean-centered or standardized, and whether latent-variable interaction or observed/composite scores were used.

 A statistically significant negative interaction coefficient alone is insufficient for interpreting moderation. The authors should present a simple-slope analysis and preferably an interaction plot showing the relationship between political literacy and political participation at low, medium, and high levels of post-truth politics.

 There is also an interpretational problem in the Results section. The manuscript states that the negative moderation result means:

 “the higher the political literacy, the weaker is the influence of post-truth politics on Gen-Z's political participation.”

 This is not the same proposition as the stated hypothesis. H5 proposes that **post-truth politics weakens the effect of political literacy on political participation**. The direction of conditionality matters. The current interpretation should therefore be corrected.

 Additionally, the manuscript states that all hypotheses were supported because the paths had “positive and significant coefficients,” although H5 has a **negative coefficient (−0.21)**. This statement is internally inconsistent and should be corrected.

 For the mediation analysis, reporting only an indirect coefficient and p-value is also inadequate. The authors should report the bootstrap confidence interval for the indirect effect. The authors should additionally clarify whether mediation is partial or full, because the direct DD → PP path remains statistically significant.

 The manuscript should also provide standardized coefficients, standard errors, critical ratios or confidence intervals for all structural paths rather than only coefficients and p-values.

 Finally, discriminant validity between the four constructs should be demonstrated. AVE establishes convergent validity but does not establish discriminant validity.

**5. Availability of underlying data — Yes**

 This is one of the stronger aspects of the manuscript. The authors state that anonymized underlying survey data are publicly available through Figshare and provide a DOI. They also provide the questionnaire as extended data.

 Therefore, I would answer **Yes** to the question regarding availability of source data.

 For maximum reproducibility, however, the authors could further improve the repository by providing the exact AMOS model/input file, variable coding documentation, derived variables/interaction terms, and ideally analysis syntax or a detailed analysis protocol. Public availability of raw data alone does not necessarily allow exact reproduction of every analytical decision.

**6. Conclusions — Partly**

 The central conclusions are generally consistent with the direction of the reported structural coefficients: digital democracy is positively associated with political literacy and political participation; political literacy is positively associated with political participation and statistically mediates the DD–PP relationship; and the interaction involving post-truth politics is negative.

 However, the strength of some conclusions exceeds what can be established from the research design. In particular, statements implying that digital democracy “strengthens” political literacy or that political literacy “enables” particular political behaviors should be expressed more cautiously because the design is observational and cross-sectional.

 Likewise, conclusions such as political literacy allowing respondents to “avoid misinformation and disinformation” appear stronger than the reported SEM results can directly establish unless this behavior was explicitly measured.

 The authors appropriately acknowledge that their quantitative approach does not capture longitudinal changes and recommend longitudinal research. However, the limitations section should additionally acknowledge retrospective recall bias, self-report bias, common-method variance, uncertainty regarding probability sampling/generalizability, and the inability of a cross-sectional design to support causal inference.

**Major points that must be addressed for scientific soundness**

 In my assessment, the manuscript could become indexable, but the following points should be addressed before approval:

1. **Resolve the sampling inconsistency of 34 versus 38 provinces and describe the respondent-selection process sufficiently to substantiate the claim of proportionate stratified sampling.**
2. **Clarify the temporal design.** Data collected in August–September 2025 are being used to make claims about participation in the 2024 election. The authors should explain exactly what respondents were asked to recall and explicitly acknowledge potential recall bias.
3. **Provide substantially greater detail on measurement development and validation**, including scale sources, item adaptation, factor loadings, CFA results, discriminant validity, and procedures for instrument validation.
4. **Fully describe and re-report the moderation analysis**, including interaction construction, centering/standardization, simple slopes, and an interaction figure. The interpretation of the negative interaction coefficient must also be corrected.
5. **Report mediation using bootstrap confidence intervals** and specify whether political literacy represents partial or full mediation.
6. **Avoid causal claims that are not warranted by a cross-sectional observational design.** Where appropriate, “influences” or “causes” should be replaced by language such as “is associated with” or “is positively related to.”
7. **Provide sufficient SEM diagnostics and analytical information**, including estimation method, normality assessment, missing-data handling, outlier assessment, standardized coefficients with uncertainty measures, and potential common-method bias.
8. **Expand the limitations section** to include self-report bias, retrospective recall bias, common-method variance, sampling/generalizability issues, and the inability to establish causality.

Subject to satisfactory revision of these points, the manuscript could make a useful contribution to research on digital democracy and youth political participation in Indonesia.

Is the work clearly and accurately presented and does it cite the current literature?

Partly

If applicable, is the statistical analysis and its interpretation appropriate?

Partly

Are all the source data underlying the results available to ensure full reproducibility?

Yes

Is the study design appropriate and is the work technically sound?

Partly

Are the conclusions drawn adequately supported by the results?

Partly

Are sufficient details of methods and analysis provided to allow replication by others?

Partly

Reviewer Expertise:

Research methodology, quantitative research, Structural Equation Modeling (SEM), organizational and social science research, digital governance, political participation, public policy, and digital transformation. I am particularly able to assess research design, measurement models, hypothesis development, mediation and moderation analysis, and the interpretation of quantitative findings.

I confirm that I have read this submission and believe that I have an appropriate level of expertise to confirm that it is of an acceptable scientific standard, however I have significant reservations, as outlined above.

# Response

**Published:** 2026-09-19

**Authors:** Agust Jovan Latuconsina

**Affiliations:** Universitas Airlangga Departemen Politik, Surabaya, East Java, Indonesia

Dear Reviewer,

 Thank you for your careful and constructive comments on our manuscript. We have substantially revised the article in response to your recommendations. The Introduction and theoretical framework were streamlined and strengthened to clarify the theoretical gap, positioning Political Literacy as an explanatory mechanism linking Digital Democracy with Political Participation and Post-Truth Politics as a contextual boundary condition. The sampling information has been corrected and standardized throughout the manuscript to reflect the final dataset of 500 respondents from all 38 provinces of Indonesia, using 77 substantive indicators. We also clarified that data were collected in August–September 2025 and that responses referring to the 2024 election were retrospective, with potential recall bias now explicitly acknowledged.

 The Methods and Results sections were expanded to provide clearer information on instrument validation, CFA, reliability, AVE, HTMT, Maximum Likelihood estimation, normality, multivariate outliers, multicollinearity, common-method bias, bootstrapping, and confidence intervals. The mediation analysis now reports a significant indirect association through Political Literacy (β = 0.192, 95% CI [0.144, 0.244]) and identifies it as partial mediation because the direct Digital Democracy–Political Participation association remained significant. The moderation analysis was also fully revised. Political Literacy and Post-Truth Politics were standardized before constructing the interaction term, and the negative interaction (β = −0.173, p < .001) is now correctly interpreted as indicating that Post-Truth Politics weakens the positive association between Political Literacy and Political Participation. Simple-slope analysis and an interaction figure have also been added.

 In addition, causal wording was systematically replaced with association-based language appropriate for the cross-sectional design. The Discussion and Conclusions were shortened and revised to avoid overstatement, while the Limitations section now addresses retrospective recall bias, self-report bias, potential common-method variance, non-probability sampling, generalizability, and the inability to establish causality. We sincerely appreciate the reviewer’s comments, which have helped us substantially improve the theoretical clarity, methodological transparency, statistical reporting, and overall rigor of the manuscript.
